# Neural Networks for Estimating Attitude, Line of Sight, and GNSS Ambiguity Through Onboard Sensor Fusion

**DOI:** 10.3390/s25237212

**Published:** 2025-11-26

**Authors:** Raul de Celis, Luis Cadarso

**Affiliations:** Aerospace Systems and Transport Research Group, Rey Juan Carlos University, 28942 Madrid, Spain; luis.cadarso@urjc.es

**Keywords:** sensor fusion, deep learning, neural estimators, GNC systems, GNSS ambiguity

## Abstract

Accurate estimation of attitude, line of sight (LOS), and carrier-phase ambiguity is essential for the performance of Guidance, Navigation, and Control (GNC) systems operating under highly dynamic and uncertain conditions. Traditional sensor fusion and filtering methods, although effective, often require precise modeling and high-grade sensors to maintain robustness. This paper investigates a deep learning-based estimation framework for attitude, LOS, and GNSS ambiguity through the fusion of onboard sensors—GNSS, IMU, and semi-active laser (SAL)—and remote sensing information. Two neural network estimators are developed to address the most critical components of the navigation chain: GNSS carrier-phase ambiguity and gravity-vector reconstruction in the body frame, which are integrated into a hybrid guidance and navigation scheme for attitude and LOS determination. These learning-based estimators capture nonlinear relationships between sensor measurements and physical states, improving generalization under degraded conditions. The proposed system is validated in a six-degree-of-freedom (6-DoF) simulation environment that includes full aerodynamic modeling of artillery guided rockets. Comparative analyses demonstrate that the learning-based ambiguity and gravity estimators reduce overall latency, enhance estimation accuracy, and improve guidance precision compared to conventional networks. The results suggest that deep learning-based sensor fusion can serve as a practical foundation for next-generation low-cost GNC systems, enabling precise and reliable operation in scenarios with limited observability or sensor degradation.

## 1. Introduction

Accurate and robust state estimation lies at the core of modern Guidance, Navigation, and Control (GNC) systems. In highly dynamic flight environments—such as spin-stabilized artillery rockets, guided munitions, and small unmanned aerial vehicles (UAVs)—the ability to determine the platform’s attitude, line of sight (LOS), and GNSS carrier-phase ambiguity is fundamental to maintaining stable control, ensuring target tracking, and achieving mission success. Each of these estimation tasks is intrinsically nonlinear and sensitive to sensor noise, modeling errors, and environmental degradation. Over the past several decades, a variety of classical approaches have been developed to address these challenges, relying on integrated INS/GNSS navigation and observer-based filtering techniques [1,2,3]. While these methods are mathematically elegant and computationally efficient, they depend heavily on accurate system models and high-grade inertial sensors, which limits their applicability in low-cost or resource-constrained platforms.

The continuous evolution of artificial intelligence and machine learning—particularly deep neural networks (DNNs)—has recently enabled the development of data-driven estimation frameworks capable of learning complex nonlinear relationships directly from multisensor data [4,5,6,7]. Neural architectures, ranging from multilayer perceptrons to recurrent and convolutional models, have shown a strong capacity to infer latent physical relationships in the presence of uncertainty. In aerospace applications, these models can approximate the dynamics of flight systems and fuse information from heterogeneous sensors, compensating for missing data or modeling inaccuracies. Reinforcement learning approaches have even demonstrated the ability to optimize guidance strategies and control laws for intercepting maneuvering targets under partially observable conditions [8,9]. These results suggest that learning-based estimation has the potential to complement or even replace traditional deterministic algorithms in demanding GNC contexts.

Throughout this work, the full projectile dynamics and guidance environment are modeled using a six-degree-of-freedom simulation framework implemented in MATLAB/Simulink R2024b, which incorporates aerodynamic, kinematic, and actuator subsystems. This environment provides the synthetic data required to train and validate the different components of the architecture. All neural networks are trained using the TensorFlow v2.16 framework with the Keras API, ensuring reproducibility and efficiency.

Attitude estimation is one of the most critical components in any GNC architecture. Conventional techniques combine inertial sensor integration with GNSS measurements in complementary or Kalman-based filters [10,11,12,13,14]. Although effective under nominal conditions, these methods are vulnerable to accumulated drift, bias instability, and measurement dropout. In contrast, deep neural networks trained on synchronized GNSS/IMU data can learn the underlying nonlinear mapping between raw signals and the corresponding orientation or gravity vector, eliminating the need for precise analytical models. Several studies have shown that fusing accelerometer data with GNSS observables enables robust attitude estimation even in the absence of high-grade gyroscopes, thereby reducing overall system cost and complexity. This is particularly advantageous for guided projectiles and small UAVs, where size, weight, and power constraints limit the onboard sensor suite.

In parallel, the estimation of the line-of-sight (LOS) vector plays a central role in terminal guidance systems employing semiactive laser (SAL) seekers. Classical methods reconstruct the LOS from geometric relationships between the projectile, the seeker, and the target’s laser spot [15,16,17,18]. However, in rapidly spinning projectiles or under turbulent flight conditions, nonlinear aerodynamic effects and sensor misalignment introduce significant estimation errors. Hybrid schemes integrating analytical guidance laws with neural correction modules have demonstrated improved performance by compensating for unmodeled dynamics and optical distortions [19,20,21]. These methods extend the operational envelope of SAL-based systems, allowing reliable target tracking at high spin rates and varying Mach numbers. In addition, neural LOS estimators can operate with partial signal information, enhancing robustness when the photodiode array experiences occlusion or degraded signal-to-noise ratio.

Beyond attitude and LOS, GNSS carrier-phase ambiguity resolution remains a persistent challenge for achieving centimeter-level accuracy in real-time navigation and attitude determination. The well-known Least-Squares Ambiguity Decorrelation Adjustment (LAMBDA) algorithm and its derivatives have established the mathematical foundation for integer ambiguity fixing [22,23]. Subsequent research has explored triple-frequency GNSS combinations to accelerate convergence and enhance precision [24,25], as well as Bayesian and multi-frequency extensions that exploit statistical prior knowledge [26,27] and an innovative NRTK framework for mobile platforms [28]. However, these algorithms are often sensitive to multipath effects, measurement outliers, and low signal-to-noise conditions. Recent work has proposed convolutional and recurrent neural networks to model the nonlinear dependencies among code and carrier-phase observables, thus improving ambiguity resolution in real time under degraded environments [29,30,31]. These neural solutions provide adaptive capabilities that classical estimators inherently lack, enabling faster ambiguity convergence without compromising accuracy.

The use of high-fidelity aerodynamic modeling and six-degree-of-freedom (6-DoF) simulations has been essential for validating such estimation and guidance frameworks. Nonlinear projectile models incorporating aerodynamic coefficients dependent on Mach number, spin rate, and angle of attack allow researchers to assess the behavior of estimators under realistic conditions [32,33,34]. These simulation environments can emulate cross-coupling between rotational and translational dynamics, actuator saturation, and time-varying external disturbances such as crosswinds or gravity anomalies. Moreover, the inclusion of complete actuator models (e.g., nose-mounted canards) permits the evaluation of guidance and control laws in closed-loop conditions, ensuring that estimator errors translate meaningfully into guidance performance metrics such as miss distance or Circular Error Probable (CEP).

While these studies have significantly advanced individual estimation domains, a notable gap remains: most existing works treat attitude, LOS, and GNSS ambiguity as separate problems handled by independent modules. In classical system architectures, attitude and LOS are recovered from inertial and SAL or optical sensors, whereas GNSS ambiguity resolution is performed by dedicated algorithms, each with its own data pipeline, filtering scheme, and computational budget. This separation often leads to redundant sensor processing, delays in information propagation, and limited exploitation of the correlations that naturally exist between the underlying quantities. For instance, accurate attitude estimation stabilizes the coordinate transformations required for LOS reconstruction, and robust ambiguity resolution provides consistent GNSS baselines for orientation determination. In this work we do not attempt to replace the entire chain with a single monolithic network; instead, we focus on two key bottlenecks—gravity vector reconstruction and GNSS double-difference ambiguity estimation—and study how learning-based estimators for these tasks, when embedded in a unified 6-DoF navigation and guidance framework, impact the overall attitude and LOS restitution performance.

In summary, this work proposes and assesses a hybrid learning-based navigation framework in which two neural estimators replace classical gyroscopic instrumentation and analytical ambiguity resolution within a realistic GNC chain for spin-stabilized projectiles. A gravity vector network and a GNSS double-difference ambiguity network are designed, trained on data generated by a high-fidelity 6-DoF simulation, and embedded in the full guidance and attitude-reconstruction pipeline. The paper reports a structured, multi-phase experimental campaign that compares classification and regression formulations, MLP and RNN architectures, time-window lengths, and training hyperparameters, providing practical design guidelines for similar aerospace estimation problems. End-to-end Monte Carlo simulations show that the proposed learning-based estimators achieve attitude restitution accuracy close to RTK-like performance while relying on lower-cost sensing and without explicit analytical ambiguity resolution. By integrating standard deep learning models into a physics-consistent 6-DoF testbed and quantifying their impact at system level, the work advances the application of neural estimation techniques to low-cost, high-dynamic GNC systems.

The proposed system is validated in a high-fidelity 6-DoF simulation environment representative of spin-stabilized projectiles, including nonlinear aerodynamics and realistic GNSS, IMU and SAL sensor models. The gravity vector and GNSS double-difference ambiguity networks are trained on synthetic datasets generated from this environment and subsequently embedded in the full guidance and attitude-restoration chain. Model performance is evaluated through Monte Carlo campaigns, including cases with degraded GNSS availability, SAL noise corruption and sensor misalignments, analysing ambiguity accuracy, gravity reconstruction error and the resulting attitude and LOS restitution. The experimental study compares MLP and RNN architectures, classification- and regression-based formulations for ambiguity, and different input time windows, and includes a reference RTK control simulation as a classical baseline. The results show that the learning-based estimators—in particular the classification-based ambiguity networks combined with the learned gravity estimator and SAL information in the terminal phase—can significantly improve ambiguity resolution robustness and gravity vector reconstruction, leading to attitude restitution RMSEs close to those of the RTK baseline while relying on lower-cost sensing and enhancing guidance performance in challenging flight conditions.

The framework provides qualitative benefits in terms of scalability and integration. By unifying multiple estimation tasks within a single network, the architecture simplifies onboard implementation and facilitates adaptive reconfiguration. The shared backbone can be extended to incorporate additional sensing modalities—such as radar or infrared cameras—without significant architectural modifications. Furthermore, the model’s differentiable nature enables end-to-end optimization with respect to downstream control and guidance objectives, opening pathways toward fully learned GNC systems.

**Figure 1:**
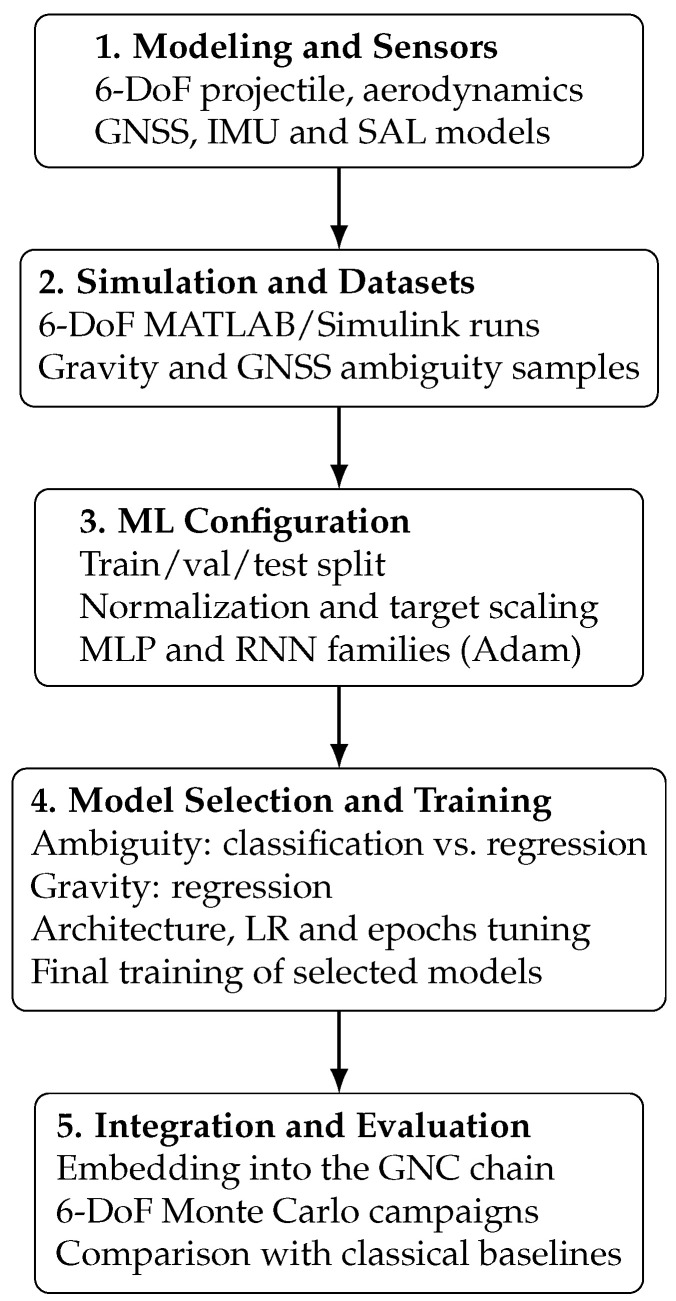
Compact workflow of the proposed learning-based framework, from modeling and dataset generation to model selection, training, and integration into the GNC system.

The remainder of this article is structured as follows. Section 2 presents the mathematical formulation underlying the projectile dynamics, sensor models, and the estimation problem. Section 3 introduces the proposed neural network architecture, detailing the network design, training strategy, and loss functions used for joint attitude, LOS, and GNSS ambiguity estimation. Section 4 describes the complete simulation environment and experimental setup, including the MATLAB/Simulink R2024b 6-DoF model and the procedures used to generate the training and validation datasets and reports the results of the Monte Carlo campaigns and compares the performance of the proposed framework against classical baselines. Finally, Section 5 summarizes the main contributions and discusses potential avenues for future research. To provide a clear overview of the methodology and to help readers navigate the transition from the theoretical formulation to the experimental implementation, Figure 1 presents the complete workflow of the proposed framework. The diagram summarizes all major steps of the study, from the mathematical modeling and dataset generation to the machine-learning procedure, model selection, and final integration within the GNC system.

## 2. Mathematical Formulation

This section presents the formal mathematical foundation of the proposed neural network based estimation framework. The formulation integrates the physical models of projectile dynamics, the sensor measurement equations, and the data-driven learning structure used to jointly estimate attitude, line of sight (LOS), and GNSS ambiguity. All vector quantities are expressed in $R^{3}$, unless otherwise stated, and follow standard aerospace notation conventions [21,22,32].

### 2.1. Characterization of the Aerial Platform

**Figure 2:**
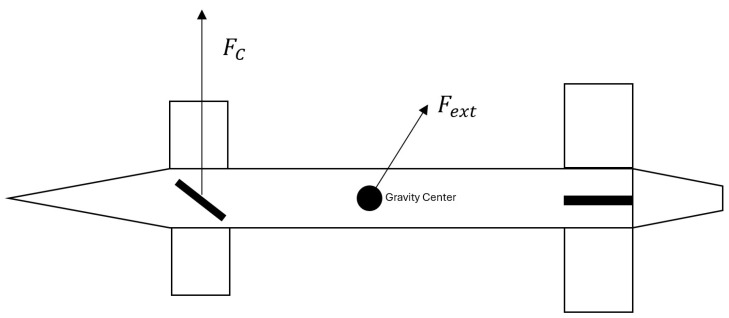
Scheme of the rocket vehicle.

The proposed GNC strategy is implemented on a guided rocket equipped with a maneuvering assembly consisting of four forward-mounted canard control surfaces. These canards are mechanically isolated from the main airframe, allowing independent roll control of the forebody. A canard, in aeronautical terms, refers to a small lifting surface located ahead of the main wing, primarily intended to enhance stability and maneuverability, often substituting the conventional horizontal tail. In this configuration, the canard pairs are operated in a differential manner—two by two—to generate the desired control forces and the corresponding aerodynamic moments required for attitude regulation. Figure 2 clarifies the configuration of the vehicle. The forces depicted in the figure are described later in this section.

Table 1 and Table 2 summarize the principal physical and aerodynamic properties of the vehicle, including thrust characteristics, total and propellant mass, inertia tensor components, and aerodynamic coefficients. These data were derived from a combination of computational fluid dynamics (CFD) analyses, experimental testing, and validation through wind tunnel campaigns (see [32] for additional details). To ensure smoothness and continuity in both thrust and aerodynamic coefficient variations, cubic-spline interpolation was applied at intermediate data points. Based on the reported inertia distribution, the vehicle exhibits symmetry about its principal planes.

### 2.2. Equations Governing the Flight Dynamics

To formulate the flight dynamics model, three reference frames are introduced for projecting forces and moments: the North–East–Down (NED) frame and the body-fixed frame.

The NED frame, denoted by the subscript *e*, is an Earth-fixed coordinate system in which the $x_{e}$ axis points toward geographic north, the $z_{e}$ axis is perpendicular to the Earth’s surface and directed downward (nadir), and the $y_{e}$ axis completes a right-handed, clockwise triad.

The body-fixed frame, denoted by the subscript *b*, is attached to the vehicle itself. The $x_{b}$ axis points forward along the longitudinal symmetry plane, $z_{b}$ is perpendicular to $x_{b}$ and directed downward within the same plane, and $y_{b}$ completes a right-handed coordinate system. The origin of this frame coincides with the vehicle’s center of gravity, and the entire triad is rigidly attached to the roll-decoupled control assembly.

The following section presents the governing equations of flight dynamics. Assuming the vehicle behaves as a rigid body, its motion is described by the classical Newton–Euler formulation. These six coupled differential equations describe both translational and rotational motion of the center of gravity as a function of the total external forces and moments acting on the body. The resultant aerodynamic, thrust, and gravitational contributions are expressed in Equations (1) and (2).

(1) $$F_{\mathrm{ext}}=F_{L}+F_{D}+F_{T}+F_{Mgn}+F_{P_{d}}+F_{G}+F_{Cor},$$

(2) $$M_{\mathrm{ext}}=M_{P_{d}}+M_{Ovr}+M_{Mgn}+M_{S_{d}}.$$

where: $F_{L}$ is the lift force, $F_{D}$ the drag force, $F_{T}$ the thrust, $F_{Mgn}$ the Magnus force, $F_{P_{d}}$ the pitch damping contribution, $F_{G}$ the gravitational force, and $F_{Cor}$ the Coriolis term.

Similarly, $M_{P_{d}}$ denotes the pitch damping moment, $M_{Ovr}$ the overturning moment, $M_{Mgn}$ the Magnus moment, and $M_{S_{d}}$ the spin damping moment.

The total external force vector $F_{\mathrm{total}}$ and moment vector $M_{\mathrm{total}}$ summarize all aerodynamic, gravitational, and inertial effects acting on the vehicle at each instant of flight. These terms are projected into the selected reference frame for integration within the full Newton–Euler dynamic model.

The external forces expressed in the Earth frame are nonlinear functions of the vehicle’s motion variables—such as aerodynamic velocity, total angle of attack, and Mach number—as well as of the aerodynamic coefficients and atmospheric properties. Due to the strong nonlinear coupling between these quantities, the explicit analytical formulation is omitted here; instead, their functional dependencies are presented. Additional mathematical details can be found in [32].

The lift force in the Earth frame is defined in Equation (3) as a nonlinear function of the angle of attack α, velocity vector $v_{e}$, and Mach number *M*:

(3) $$F_{L}^{\left(e\right)}=F_{L}^{\left(e\right)}\left(\alpha ,x_{e},v_{e},M,\rho ;C_{L1},C_{L3},S\right).$$

The drag force, shown in Equation (4), depends primarily on α, Mach number, and air density:

(4) $$F_{D}^{\left(e\right)}=F_{D}^{\left(e\right)}\left(\alpha ,v_{e},M,\rho ;C_{D0},C_{D2},S\right).$$

The pitch damping contribution, given in Equation (5), is influenced by angular momentum and moment of inertia:

(5) $$F_{P_{d}}^{\left(e\right)}=F_{P_{d}}^{\left(e\right)}\left(x_{e},v_{e},H_{e},M,\rho ;C_{N_{q}},I_{y},S\right).$$

The Magnus force, presented in Equation (6), depends on spin rate and cross-coupling effects:

(6) $$F_{Mgn}^{\left(e\right)}=F_{Mgn}^{\left(e\right)}\left(x_{e},v_{e},H_{e},M,\rho ;C_{M_{f}},I_{x},S\right).$$

The thrust contribution is modeled in Equation (7), as a function of the thrust direction vector $x_{e}$:

(7) $$F_{T}^{\left(e\right)}=F_{T}^{\left(e\right)}\left(x_{e}\right).$$

The gravitational force is represented by Equation (8), which depends on the gravitational acceleration vector $g_{e}$ and the vehicle mass *m*:

(8) $$F_{G}^{\left(e\right)}=F_{G}^{\left(e\right)}\left(g_{e};m\right).$$

Finally, Equation (9) expresses the Coriolis contribution, which arises from the Earth’s angular velocity Ω and the relative motion of the vehicle:

(9) $$F_{Cor}^{\left(e\right)}=F_{Cor}^{\left(e\right)}\left(v_{e};m,\Omega \right).$$

Here, $C_{L1}$ and $C_{L3}$ denote the linear and cubic lift coefficients, respectively, while α represents the total angle of attack. The coefficients $C_{D0}$ and $C_{D2}$ correspond to the drag model (linear and quadratic terms). The vector $H_{e}$ is the angular momentum expressed in the Earth frame, and $I_{x}$, $I_{y}$ are the principal moments of inertia in the body frame. $C_{N_{q}}$ is the pitch damping coefficient, and $C_{M_{f}}$ the Magnus force coefficient. The vector $x_{e}$ represents the vehicle pointing direction, $g_{e}$ the gravity vector in the Earth frame, Ω the Earth’s rotation vector, and $v_{e}$ the velocity of the vehicle in the same frame. Finally, *S* denotes the reference aerodynamic surface and ρ the local air density.

Similarly, the aerodynamic and inertial moments acting on the vehicle in the Earth frame are given by the following expressions. These include the contributions of overturning, pitch damping, Magnus, and spin damping effects, all of which depend nonlinearly on the vehicle’s dynamic state, aerodynamic parameters, and flow conditions.

The pitch damping moment, defined in Equation (10), depends on the vehicle’s angular motion and the coefficient $C_{M_{q}}$:

(10) $$M_{P_{d}}^{\left(e\right)}=M_{P_{d}}^{\left(e\right)}\left(x_{e},v_{e},H_{e},M,\rho ;C_{M_{q}},I_{y},S\right).$$

The overturning moment is described in Equation (11), where $C_{M1}$ and $C_{M3}$ represent the linear and cubic coefficients:

(11) $$M_{Ovr}^{\left(e\right)}=M_{Ovr}^{\left(e\right)}\left(\alpha ,x_{e},v_{e},M,\rho ;C_{M1},C_{M3},S\right).$$

The Magnus moment, expressed in Equation (12), accounts for rotational flow coupling:

(12) $$M_{Mgn}^{\left(e\right)}=M_{Mgn}^{\left(e\right)}\left(x_{e},v_{e},H_{e},M,\rho ;C_{mm},I_{x},S\right).$$

The spin damping moment, shown in Equation (13), represents the stabilizing torque due to roll damping:

(13) $$M_{S_{d}}^{\left(e\right)}=M_{S_{d}}^{\left(e\right)}\left(x_{e},v_{e},H_{e},M,\rho ;C_{spin},I_{x},S\right).$$

Here, $C_{M_{q}}$ denotes the pitch damping moment coefficient, while $C_{M1}$ and $C_{M3}$ are the linear and cubic overturning moment coefficients, respectively. The coefficient $C_{mm}$ corresponds to the Magnus moment contribution, and $C_{spin}$ represents the spin damping moment coefficient. The variables $x_{e}$ and $v_{e}$ describe the position and velocity vectors in the Earth frame, $H_{e}$ is the angular momentum, *M* the Mach number, ρ the air density, and *S* the vehicle’s reference surface. The principal moments of inertia $I_{x}$ and $I_{y}$ are included to account for the coupling between translational and rotational dynamics in the roll-decoupled configuration.

The control forces and moments, generated by the maneuvering system composed of four independently actuated canards, are expressed in Equations (14) and (15). These nonlinear relations depend on the aerodynamic flow, canard deflection, and local orientation of each surface.

(14) $$F_{C}=\sum_{i=1}^{4}F_{C_{i}}\left(\alpha ,x_{b},v_{e},M,\rho ,\delta_{i},n_{i};C_{N_{e}},S,S_{c}\right),$$

(15) $$M_{C}=\sum_{i=1}^{4}M_{C_{i}}\left(\alpha ,x_{b},v_{e},M,\rho ,\delta_{i},n_{i};C_{N_{e}},S,S_{c}\right).$$

Here, $x_{b}$ is the forward-pointing unit vector lying in the vehicle’s plane of symmetry, while $\delta_{i}$ denotes the deflection angle of the *i*-th canard. The vector $n_{i}$ represents the surface normal to the corresponding canard aerodynamic plane. The term $C_{N_{e}}$ is the aerodynamic coefficient associated with the normal force on each fin, *S* is the vehicle reference area, and $S_{c}$ denotes the characteristic surface area of each canard element. The total control force $F_{C}$ and moment $M_{C}$ are obtained by summing the individual contributions of the four actuators.

As previously stated, a Newton–Euler formulation is adopted to describe the equations of motion of the vehicle. The dynamics are expressed in both the body-fixed frame (denoted as *b*) and the flat-Earth frame (denoted as *e*). These frames are related through the Euler angles of yaw (ψ), pitch (θ), and roll (ϕ), which define the vehicle’s orientation in space. The complete formulation is presented in Equations (16) and (17).

The translational motion of the center of mass, expressed in the body-fixed reference frame, follows Newton’s second law as shown in Equation (16):

(16) $$F_{C}^{\left(b\right)}+F_{\mathrm{ext}}^{\left(b\right)}=\frac{d}{dt}\left(m\;v_{b}\right)+\omega_{b}\times \left(m\;v_{b}\right),$$

where $v_{b}$ represents the linear velocity of the vehicle in body axes, $\omega_{b}$ is the angular velocity vector, and *m* is the total mass of the system. The term $\omega_{b}\times \left(m\;v_{b}\right)$ accounts for the Coriolis effects arising from the rotation of the body frame.

Similarly, the rotational motion of the vehicle is governed by Euler’s equation, shown in Equation (17):

(17) $$M_{C}^{\left(b\right)}+M_{\mathrm{ext}}^{\left(b\right)}=\frac{dL_{b}}{dt}+\omega_{b}\times L_{b},$$

where $L_{b}$ is the angular momentum vector expressed in body axes, defined as $L_{b}=I_{b}\;\omega_{b}$, with $I_{b}$ being the inertia tensor referred to the body frame. The cross product $\omega_{b}\times L_{b}$ represents the gyroscopic coupling between roll, pitch, and yaw dynamics.

Equations (16) and (17) together constitute the six-degree-of-freedom (6-DoF) Newton–Euler equations of motion. To ensure consistency, all control forces and moments ($F_{C}^{\left(b\right)}$, $M_{C}^{\left(b\right)}$) as well as external forces and moments ($F_{\mathrm{ext}}^{\left(b\right)}$, $M_{\mathrm{ext}}^{\left(b\right)}$) must be expressed in the body-fixed coordinate system prior to their inclusion in these dynamic equations.

### 2.3. Attitude Determination Equations

The vehicle’s attitude can be geometrically characterized by identifying two non-collinear direction vectors, denoted here as $a_{1}$ and $a_{2}$, expressed in two distinct reference frames—for example, the conventional Earth-fixed North–East–Down (NED) frame and the body-fixed coordinate system.

These two direction vectors, $a_{1}$ and $a_{2}$, span a unique plane in three-dimensional space. The orientation of this plane is determined by its associated normal vector, $a_{3}$, which is computed as the vector cross product of $a_{1}$ and $a_{2}$, as defined in Equation (18).

(18) $$a_{3}=a_{1}\times a_{2}.$$

The transformation between the Earth reference frame and the body-fixed coordinate system can be expressed through a rotation tensor, denoted as $T_{eb}$. For convenience and numerical stability, each direction vector $a_{i}$ (i=1,2,3) is normalized according to $n_{i}=a_{i}/∥a_{i}∥$. The rotation tensor can then be assembled following Equation (19), where the normalized direction vectors $n_{i}$ are arranged as column components.

This approach is fundamental in determining the spatial orientation of flight vehicles, as it allows the precise mapping between the Earth and body axes. By employing a rotation tensor, onboard sensors can be correctly aligned with the vehicle’s reference frame, thereby ensuring accurate measurement interpretation. Such information is essential for the guidance, navigation, and control (GNC) system, allowing the onboard computer to continuously correct deviations and maintain the desired trajectory. Overall, this method provides a reliable and computationally efficient framework for attitude estimation in aerospace systems.

(19) $$T_{eb}=\left[\begin{array}{ccc} n_{1_{e}} & n_{2_{e}} & n_{3_{e}} \end{array}\right]\cdot {\left[\begin{array}{ccc} n_{1_{b}} & n_{2_{b}} & n_{3_{b}} \end{array}\right]}^{-1}$$

The use of multiple vectors instead of only three provides redundancy and significantly increases the robustness of the attitude estimation. The number of sensors employed in this study to provide the sources for those vectors, was selected based on a trade-off between computational cost and accuracy. Increasing the number of sorces improves the geometric conditioning of the rotation matrix but also increases the system complexity.

Consequently, Equation (19) can be generalized to accommodate an arbitrary number of direction vectors, as expressed in Equation (20).

(20) $$T_{eb}={\left(N_{e}N_{e}^{T}\right)}^{-1}N_{e}N_{b}^{T},\;where\;N_{\mathrm{frame}}={\left[\begin{array}{ccc} n_{1} & \cdots & n_{N-1} \end{array}\right]}_{\mathrm{frame}},\;\mathrm{frame}\in \left\{e,b\right\}.$$

The next subsections outline two complementary methods for deriving the required vector pairs.

#### 2.3.1. Velocity, Line of Sight and Gravity Vector Method

As mentioned earlier, determining the vehicle’s attitude requires identifying at least two distinct vectors expressed in two different reference frames. In this work, the velocity vector and the line-of-sight (LOS) vector are used as the reference pair.

If a GNSS sensor suite is installed on the vehicle, the velocity vector can be directly obtained from GNSS measurements expressed in the Earth-fixed frame (denoted by subscript *e*). The velocity components in this frame, namely $v_{x_{e}}$, $v_{y_{e}}$, and $v_{z_{e}}$, define the velocity vector as shown in Equation (21).

(21) $$v_{e}={\left[v_{x_{e}},\;v_{y_{e}},\;v_{z_{e}}\right]}^{T}$$

In parallel, the same velocity vector can also be computed in the body-fixed frame from a triad of accelerometers, one mounted along each principal axis. By integrating their measured accelerations over time, the velocity vector in the body frame is obtained as expressed in Equation (22). In this equation, $a_{x_{b}}$, $a_{y_{b}}$, and $a_{z_{b}}$ denote the acceleration components measured along the body axes, and ${\tilde{\omega }}_{b}$ represents the estimated angular velocity vector, also expressed in body coordinates. It should be noted that, at this stage, ${\tilde{\omega }}_{b}$ remains unknown; the algorithm used to estimate it will be presented in the following sections.

(22) $$v_{b}=\int \left\{{\left[a_{x_{b}},\;a_{y_{b}},\;a_{z_{b}}\right]}^{T}+{\tilde{\omega }}_{b}\times v_{b}\right\}dt$$

Similarly, the line-of-sight (LOS) vector must be determined in both the Earth-fixed and body-fixed reference frames, denoted as ${\mathrm{LOS}}_{e}$ and ${\mathrm{LOS}}_{b}$, respectively. The vector ${\mathrm{LOS}}_{e}$ can be readily obtained from GNSS sensor data, as it directly provides the vehicle and target positions in Earth coordinates. However, determining ${\mathrm{LOS}}_{b}$ requires the use of the semi-active laser (SAL) seeker, which becomes operational only when the vehicle is sufficiently close to the target. Consequently, during the initial and mid-course flight phases, when the SAL signal is unavailable, an additional reference vector is required to enable continuous and reliable attitude estimation.

The gravity vector constitutes a natural candidate for this purpose. Determining the gravity vector in the Earth-fixed reference frame is straightforward, as it is always aligned with the $z_{e}$ axis. Its expression is given in Equation (23), where *g* denotes the gravitational acceleration, assumed constant in this model with a value of $9.81\;{m/s}^{2}$. It should be noted that higher accuracy can be achieved using more refined gravity models, in which *g* varies with latitude, longitude, and altitude.

(23) $$g_{e}=g{\left[0,\;0,\;1\right]}^{T}$$

However, the gravity vector expressed in the body-fixed reference frame, $g_{b}$, is also required. Although several methods exist to estimate $g_{b}$, most are either complex or require additional sensors. For example, one approach involves identifying the constant component of the measured acceleration using a low-pass filter. In this method, the jerk in the body frame is computed as the time derivative of the acceleration; integration of this term yields the non-constant component of the motion, which is then subtracted from the measured acceleration to isolate the gravity component. Nonetheless, this technique becomes invalid when the vehicle undergoes rotational motion.

Another classical method to estimate $g_{b}$ relies on the integration of the inertial mechanization equations [35]. However, such an approach requires gyroscopes to provide angular rate information, contradicting the low-cost, gyro-free philosophy of the proposed system.

Therefore, the cornerstone of the present attitude estimation framework lies in accurately determining the gravity vector in the body-fixed frame using only accelerometer data. The Neural Networw methodology section introduces an estimation method that remains valid for both rotating and non-rotating vehicles while relying exclusively on accelerometers.

#### 2.3.2. Array of GNSS Sensors Methodology

In the context of a vehicle equipped with an array of Global Navigation Satellite System (GNSS) antennas, the pair of vectors required for attitude determination can be directly obtained from the spatial distribution of GNSS sensors. A minimum of N=3 antennas (and up to N=8 in the tests conducted in this research) are required to reconstruct the rigid-body attitude.

The eight GNSS antennas are installed along the vehicle body as follows: the reference antenna is located at the center of mass, coinciding with the origin of the body-fixed frame. Two lateral antennas are mounted symmetrically at coordinates [0, 0.1, 0] m and [0, −0.1, 0] m in body axes. Two additional antennas are positioned along the longitudinal direction—one near the nose at [1, 0, 0] m and another near the tail at [−1, 0, 0] m. Finally, three antennas are mounted near the fin roots at [−0.8, 0.1, 0.1] m, [−0.8, −0.1, 0.1] m, and [−0.8, 0, −0.1] m. This geometric configuration provides full spatial coverage for accurate reconstruction of the orientation, while minimizing aerodynamic interference and preserving symmetry about the longitudinal axis.

Each antenna continuously transmits its GNSS position to the onboard computer. Taking one of them (denoted $a_{1}$) as the reference, the vectors connecting it to the remaining antennas are computed as $a_{a_{1},a_{j}}$ for j=2,…,N. After normalization, the following set of unit vectors is obtained in both coordinate frames:

$$n_{1}=\frac{a_{a_{1},a_{2}}}{∥a_{a_{1},a_{2}}∥},\;\ldots ,\;n_{N-1}=\frac{a_{a_{1},a_{N}}}{∥a_{a_{1},a_{N}}∥}.$$

### 2.4. Sensor Models

As outlined in the introduction, the main goal of this research is to streamline onboard navigation architectures by minimizing the reliance on complex and costly instrumentation. In conventional guidance and navigation systems, gyroscopes are typically used to measure angular velocity and orientation. Although these sensors provide accurate short-term attitude information, their performance tends to deteriorate in highly dynamic flight conditions, while maintaining precision under such regimes significantly increases system cost and complexity. To address this limitation, the proposed approach seeks to eliminate the dependency on gyroscopes by leveraging the fusion of Global Navigation Satellite System (GNSS) measurements, accelerometer data, and photodetector-based line-of-sight information. By integrating these complementary sources, the navigation solution enhances overall accuracy and robustness without the need for high-grade inertial sensors. This section presents the mathematical models used to characterize the behavior of each sensor and their role within the data fusion framework.

#### 2.4.1. GNSS Sensor Modeling

Global Navigation Satellite System (GNSS) receivers determine position and attitude by measuring the carrier phase offset, denoted as ϕ, expressed in units of the carrier wavelength λ. Under ideal conditions, this observable allows for sub-centimeter accuracy, theoretically reaching approximately 0.003 m. However, such precision requires resolving the integer phase ambiguity *N*, representing the total number of complete wavelength cycles between a satellite and a receiver antenna. This ambiguity resolution remains one of the key challenges in high-accuracy GNSS-based navigation.

Several sources of error contribute to measurement uncertainty, including the satellite clock bias $b^{S_{j}}$, the receiver clock bias $b_{a_{i}}$, propagation delays caused by the ionosphere and troposphere, $E_{atm}$, and local effects such as thermal noise and multipath interference $E_{mp}$. Combining these elements, the pseudo-range observable, $\rho_{a_{i}}^{S_{j}}$—which represents the apparent distance between satellite $S_{j}$ and receiver $a_{i}$—can be expressed as:

(24) $$\rho_{a_{i}}^{S_{j}}=c\left(t_{r_{a_{i}}}-t_{t}^{S_{j}}\right)=\lambda \left(\phi_{a_{i}}^{S_{j}}+N_{a_{i}}^{S_{j}}\right)+c\;\left(b^{S_{j}}-b_{a_{i}}\right)+E_{mp}^{S_{j},a_{i}}+E_{atm}^{S_{j},a_{i}},$$

where $t_{r_{a_{i}}}$ and $t_{t}^{S_{j}}$ denote the signal reception and transmission times, respectively, and *c* is the speed of light. Equation (24) combines deterministic components (geometric range, clock biases) and stochastic components (multipath, atmospheric delays).

In attitude determination applications, not all error terms can be explicitly estimated.

Nevertheless, since several error sources are common to all receivers, differencing techniques can be applied to mitigate or eliminate their impact. For each satellite $S_{j}$, a line-of-sight (LOS) unit vector from receiver $a_{i}$ to the satellite is defined as $e_{a_{i}}^{S_{j}}$. Given that the baseline between receivers is negligible compared to the satellite range, the LOS direction can be considered identical for all receivers and is denoted as $e^{S_{j}}$. Consequently, the relative geometry between receivers $a_{i}$ and $a_{n}$ observing the same satellite can be expressed as

(25) $$a_{a_{i},a_{n}}=\left(\rho_{a_{i}}^{S_{j}}-\rho_{a_{n}}^{S_{j}}\right)e^{S_{j}}.$$

The difference between two pseudo-range measurements from distinct receivers tracking the same satellite, known as the *single difference*, is given by (26):

(26) $$\Delta \rho_{a_{i},a_{n}}^{S_{j}}=\Delta \phi_{a_{i},a_{n}}^{S_{j}}+\Delta N_{a_{i},a_{n}}^{S_{j}}-c\cdot \Delta dT_{a_{i},a_{n}}+\Delta E_{a_{i},a_{n}}^{S_{j}}$$

The differencing operation removes common-mode errors, such as satellite clock bias and atmospheric propagation delay, which are approximately identical across closely spaced receivers.

To further suppress receiver-dependent biases, a *double difference* is formed by subtracting the single difference of one satellite, $S_{m}$, from that of another, $S_{j}$. This yields the double-differenced observable $\nabla \rho_{a_{i},a_{n}}^{S_{j},S_{m}}$, defined by (27):

(27) $$\nabla \rho_{a_{i},a_{n}}^{S_{j},S_{m}}=\nabla \phi_{a_{i},a_{n}}^{S_{j},S_{m}}+\nabla N_{a_{i},a_{n}}^{S_{j},S_{m}}+\nabla E_{a_{i},a_{n}}^{S_{j},S_{m}}$$

The receiver clock bias term cancels completely in Equation (27), which significantly improves robustness in the carrier-phase solution. However, multipath-related components, $\nabla E_{a_{i},a_{n}}^{S_{j},S_{m}}$, cannot be eliminated through differencing since they vary with antenna position and local scattering environment.

As noted by [29], multipath effects are not well modeled by white noise due to their temporal and spatial correlation. Although multipath has a substantial influence on ground or low-altitude navigation—particularly in environments with reflective surfaces such as airports—its contribution diminishes considerably during cruise flight, where the primary source of reflection is the missile body itself [36]. This assumption has been adopted in the present work.

Alternative approaches for compensating correlated GNSS errors include the use of deep learning architectures, such as recurrent or long short-term memory (LSTM) networks [29,37]. Nevertheless, given that the present study focuses on mid- and high-altitude flight, the simplified differencing model presented in Equation (27) provides a suitable trade-off between computational efficiency and estimation accuracy for the proposed navigation framework.

As detailed previously, GNSS sensors can provide highly accurate measurements of carrier phase differences, expressed through double-differenced observations. Once the integer carrier ambiguity term, $\nabla N_{a_{i},a_{n}}^{S_{j},S_{m}}$, is determined—either by estimation or by correction through a trained neural network as introduced in subsequent sections—the double-difference of pseudo-ranges, $\nabla \rho_{a_{i},a_{n}}^{S_{j},S_{m}}$, can be computed directly from carrier-phase data, as shown in Equation (27). Applying this to the previously defined geometric relationship in Equation (25), the following expression is obtained:

(28) $$\nabla \rho_{a_{i},a_{n}}^{S_{j},S_{m}}=a_{a_{i},a_{n}}\cdot \left(e^{S_{j}}-e^{S_{m}}\right),$$

where $a_{a_{i},a_{n}}$ is the baseline vector between sensors $a_{i}$ and $a_{n}$, and $e^{S_{j}}$ and $e^{S_{m}}$ are the unit vectors pointing toward satellites $S_{j}$ and $S_{m}$, respectively. This expression links the measured double-difference in carrier phase to the geometric configuration of the GNSS satellite constellation and the sensor array mounted on the vehicle.

Equation (28) can be reformulated in matrix form to simultaneously account for all available satellites, as shown in Equation (29). Here, ${\left(H^{T}H\right)}^{-1}H^{T}$ represents the Moore–Penrose pseudo-inverse of the geometry matrix *H*, which allows estimation of the baseline vector in a least-squares sense:

(29) $$a_{a_{i},a_{n}}={\left(H^{T}H\right)}^{-1}H^{T}\nabla \rho_{a_{i},a_{n}},$$

where $\nabla \rho_{a_{i},a_{n}}$ is the column vector containing the set of double-differenced pseudo-range measurements with respect to a reference satellite $S_{j}$, as defined in Equation (30), and *H* is the geometry matrix composed of the relative satellite direction cosines, as expressed in Equation (31):

(30) $$\nabla \rho_{a_{i},a_{n}}=\left[\begin{array}{c} \nabla \rho_{a_{i},a_{n}}^{S_{j},S_{p}}\\ \vdots \\ \nabla \rho_{a_{i},a_{n}}^{S_{j},S_{m}} \end{array}\right],$$

(31) $$H=\left[\begin{array}{ccc} e_{x}^{S_{j}}-e_{x}^{S_{p}} & e_{y}^{S_{j}}-e_{y}^{S_{p}} & e_{z}^{S_{j}}-e_{z}^{S_{p}}\\ \vdots & \vdots & \vdots \\ e_{x}^{S_{j}}-e_{x}^{S_{m}} & e_{y}^{S_{j}}-e_{y}^{S_{m}} & e_{z}^{S_{j}}-e_{z}^{S_{m}} \end{array}\right].$$

The vectors $e^{S_{p}}={\left[e_{x}^{S_{p}},\;e_{y}^{S_{p}},\;e_{z}^{S_{p}}\right]}^{T}$ are obtained directly from the GNSS navigation data, representing the direction from the vehicle to each satellite in the Earth-fixed reference frame. Equation (28) therefore defines an overdetermined system that can be optimally solved through Equation (29) whenever $\left(H^{T}H\right)$ is invertible. If this condition is not met, an approximate solution for the baseline vector, ${\hat{v}}_{a_{i},a_{n}}$, can be obtained by minimizing the residual norm according to

(32) $$\min_{{\hat{v}}_{a_{i},a_{n}}}{∥\nabla \rho_{a_{i},a_{n}}-H{\hat{v}}_{a_{i},a_{n}}∥}_{2}.$$

The recovered baseline vectors among all sensor pairs define the spatial geometry of the GNSS array rigidly attached to the vehicle body. By comparing these vectors expressed in the Earth-fixed frame and in the body-fixed frame, the rotation matrix relating both reference systems can be reconstructed using the same formalism introduced in Equation (29). This provides an independent and highly accurate estimation of the vehicle’s attitude without relying on gyroscopes or additional attitude sensors.

Consequently, the angular restitution approach represents a complementary and self-contained method for attitude determination, valid throughout all flight phases. When fused with accelerometer, GNSS, and semi-active laser (SAL) data, it contributes to a robust hybrid navigation system capable of maintaining accurate attitude and position estimates even under high-dynamic flight conditions.

#### 2.4.2. Accelerometers

An accelerometer measures the specific force acting on the vehicle, that is, the instantaneous rate of change of velocity within the body-fixed reference frame. In this study, each accelerometer is modeled as an ideal sensor affected by a bias and additive white noise process, with a standard deviation of approximately $0.001\;{m/s}^{2}$, which reflects the performance of typical high-grade navigation devices. The sensor model can therefore be represented as

(33) $$a_{m}=a_{b}+b_{a}+\eta_{a},$$

where $a_{m}$ denotes the measured acceleration vector in the body frame, $a_{b}$ is the true specific acceleration of the vehicle, $b_{a}$ represents the accelerometer bias (assumed to vary slowly with time), and $\eta_{a}$ is a zero-mean Gaussian noise vector.

The estimated velocity in body axes is subsequently employed, together with other onboard measurements, to determine the vehicle’s inertial velocity and orientation through sensor fusion. It is important to note that, due to the integration process, even small bias or noise components in acceleration cause cumulative drift in the velocity estimate over time. Such errors must therefore be mitigated through complementary or Kalman-based filtering strategies.

Beyond their role in kinematic state propagation, accelerometer measurements are also used for gravity vector estimation. As detailed in subsequent sections, the magnitude of the measured acceleration is exploited to infer the direction of the local gravity vector once the translational motion component has been removed. Accurate knowledge of the vehicle’s velocity magnitude is essential for this purpose, as it enables separation of inertial and gravitational contributions within the measured acceleration signal.

#### 2.4.3. Semi-Active Laser Kit

Laser-based guidance provides a reliable means of steering a vehicle toward a designated target using the reflection of a laser beam. In this approach, an external laser illuminates the target continuously, and part of the reflected radiation is scattered in multiple directions. When the vehicle enters the region where a portion of this scattered energy reaches its optical seeker, a semi-active laser (SAL) kit detects the direction of the incoming energy and generates control signals to adjust the vehicle trajectory toward the illuminated spot. This optical seeker, therefore, constitutes the primary element of the terminal guidance system.

The SAL kit employs a quadrant photo-detector composed of four independent photodiodes that convert incident light into electrical currents, denoted as $I_{1}$, $I_{2}$, $I_{3}$, and $I_{4}$. The relative distribution of these intensities defines the position of the laser spot centroid on the detector surface. Following the formulation in [19], the normalized coordinates of the centroid on the sensor plane can be estimated as

(34) $$\left[\begin{array}{c} x_{quad}\\ y_{quad} \end{array}\right]=\left[\begin{array}{c} \ln\frac{I_{4}}{I_{2}}\\ \ln\frac{I_{1}}{I_{3}} \end{array}\right].$$

The corresponding radial distance from the detector center, $r_{quad}$, can then be determined as

(35) $$r_{quad}=\sqrt{{\left(\ln\frac{I_{4}}{I_{2}}\right)}^{2}+{\left(\ln\frac{I_{1}}{I_{3}}\right)}^{2}}.$$

Although these computed coordinates are conformally related to the actual centroid position, a correction is required to obtain the true radial displacement, $r_{c}$, on the detector plane. Experimental calibration data provide the nonlinear mapping $r_{c}=f\left(r_{quad}\right)$, as shown in Table 3. A cubic spline interpolation is then applied to approximate this relationship as a continuous function.

Once the calibration function $f(r_{quad})$ is known, the corrected spot coordinates on the detector plane can be obtained using Equation (36):

(36) $$\left[\begin{array}{c} x_{c}\\ y_{c} \end{array}\right]=R_{det}\;\frac{r_{c}}{r_{quad}}\left[\begin{array}{c} \ln\frac{I_{4}}{I_{2}}\\ \ln\frac{I_{1}}{I_{3}} \end{array}\right],$$

where $R_{det}$ is the physical radius of the photo-detector. From these coordinates $\left(x_{c},y_{c}\right)$, the direction of the line-of-sight (LOS) vector can be reconstructed in the body-fixed coordinate system, considering the geometric offset between the photo-detector and the vehicle’s center of mass.

It is important to note that the SAL seeker provides useful information only during the terminal phase of the flight—when the reflected laser energy is strong enough to be detected. Nevertheless, this phase is also the most critical, as small errors in relative positioning between target and vehicle (on the order of a few meters) can cause significant impact deviations. Consequently, integrating a high-precision terminal guidance device such as a semi-active laser seeker is strongly recommended for this stage of the mission.

The fusion of the SAL sensor data with measurements from GNSS and inertial accelerometers enables an accurate estimation of the line-of-sight vector and overall vehicle attitude. This multisensor hybridization ensures precise trajectory correction during terminal homing, enhancing both hit accuracy and robustness under real flight conditions.

## 3. Guidance, Navigation and Control (GNC) Algorithm Definition

This section details the proposed navigation, guidance and control algorithms.

### 3.1. Neural Network-Based Estimation of the Gravity Vector and Carrier Phase Ambiguity

Among the numerous applications of machine learning in modern guidance, navigation, and control (GNC) systems (see [38,39,40,41,42]), its capability to infer hidden states or complex physical quantities directly from raw sensor data offers an outstanding opportunity for enhancing the accuracy and robustness of flight navigation algorithms. In particular, neural networks (NN) can be trained to learn nonlinear mappings that are difficult or even impossible to model analytically, such as the gravity vector in body coordinates or the double differences of the GNSS carrier phase ambiguity. Previous works [43,44] have shown that NNs can effectively emulate nonlinear dynamic systems and flight mechanics equations even under significant uncertainty.

The methodology presented here employs two distinct sets of neural networks, each trained with synthetic data obtained from high-fidelity simulation environments. The first network estimates the gravity vector expressed in body axes, while the second network determines the carrier phase ambiguity differences used in attitude reconstruction.

#### 3.1.1. Dataset Generation for Gravity Vector Estimation

The estimation of the gravity vector is based on the nonlinear relationship between the accelerations measured by the onboard accelerometers and the components of the true gravitational acceleration expressed in the body reference frame. To this end, a six-degree-of-freedom (6-DOF) flight dynamics simulator is employed to reproduce the behavior of a four-canard controlled aerial vehicle under various flight conditions, including steady, accelerated, and rotational maneuvers. This high-fidelity simulator provides the time history of both the true gravity vector, $g_{b}={\left[g_{x_{b}},\;g_{y_{b}},\;g_{z_{b}}\right]}^{T}$, and the accelerations measured by each onboard accelerometer, $a_{m}={\left[a_{x_{b}},\;a_{y_{b}},\;a_{z_{b}}\right]}^{T}$, as defined in Equation (33).

The neural network input corresponds to the raw acceleration data (including bias and noise), while the output target is the true gravity vector at each simulation step, as expressed in Equation (37):

(37) $${\tilde{g}}_{b}=\left[\begin{array}{c} {\tilde{g}}_{x_{b}}\\ {\tilde{g}}_{y_{b}}\\ {\tilde{g}}_{z_{b}} \end{array}\right].$$

The dataset is generated by simulating multiple flight scenarios that include climb, cruise, dive, and coordinated turns, ensuring robustness and generalization capability across a wide range of flight conditions. To emulate realistic sensor performance, Gaussian white noise with σ=0.001 m/s^2^ and a slowly varying bias are added to the accelerometer readings, replicating the behavior of navigation-grade sensors. The resulting dataset consists of 10,000 samples, each containing a 3-dimensional input vector (accelerations) and a corresponding 3-dimensional output target (gravity components). Data are randomly divided into training (70%), validation (15%), and testing (15%) subsets. Once trained, the neural network provides a high-precision estimation of the local gravity vector directly from accelerometer readings, even under rotational dynamics where conventional low-pass filtering approaches fail.

A representative subset of the training dataset is presented in Table 4, illustrating the relationship between the measured acceleration components and the true gravity vector components used as targets for network training. For clarity, only a few samples (from a total of 10,000) are shown below.

#### 3.1.2. Dataset Generation for GNSS Carrier Phase Ambiguity Estimation

In parallel, a second neural network is trained to estimate the double differences of the GNSS carrier phase ambiguity, $\nabla N_{a_{i},a_{n}}^{S_{j},S_{m}}$, which are essential for the angular restitution approach described earlier. For this purpose, an Orolia GSG-8 GNSS signal simulator [45] is employed to reproduce the satellite constellation, signal propagation, and receiver geometry for a set of eight sensors and six visible satellites. One sensor and one satellite are selected as references, while the remaining sensors and satellites are used to construct the relative measurements.

Each training sample consists of an input vector composed of the double-differenced pseudo-range and error terms $\nabla \rho_{a_{i},a_{n}}^{S_{j},S_{m}}-\nabla E_{a_{i},a_{n}}^{S_{j},S_{m}}$, and an output corresponding to the desired double-differenced carrier phase ambiguity, $\nabla N_{a_{i},a_{n}}^{S_{j},S_{m}}$. Thus, the neural network learns to infer the ambiguity differences directly from measurable GNSS quantities.

The dataset configuration corresponds to 35 input and 35 output variables, plus one additional feature describing the satellite constellation geometry. The simulation spans a 24-h period centered over the URJC campus in Madrid, with a temporal resolution of 15 s per sample (1/15 Hz). This time step ensures quasi-stationarity of the satellite configuration while capturing realistic orbital motion. To enhance statistical representativeness, 100 independent simulations are performed, introducing both random and spatially correlated noise according to the models described in [46]. The resulting dataset contains 36×576,000 input samples and 35×576,000 output samples, which can be processed efficiently on a standard workstation.

#### 3.1.3. Integration and Generalization

Both networks are trained independently and later integrated into the navigation system. The first network provides continuous estimation of the gravity vector in the body frame, while the second supplies accurate estimates of GNSS carrier phase ambiguities. Together, these estimates enhance the attitude determination process by reducing the reliance on gyroscopes and improving robustness in high-dynamic flight conditions. The methodology demonstrates the feasibility of using physics-informed neural models for hybrid navigation, paving the way for future developments in real-time adaptive flight control and autonomous guidance systems.

### 3.2. Machine Learning Methodology

Building on the concepts introduced in the preceding sections, this work investigates the application of Machine Learning (ML) methods to improve the attitude and navigation estimation of an aerospace platform without depending on conventional gyroscopic sensors. The proposed approach combines two distinct yet complementary neural network architectures, each targeting a different aspect of the overall estimation process.

The first neural model is devoted to predicting the components of the local gravity vector in the body coordinate frame, relying solely on accelerometer measurements. It learns the complex nonlinear mapping between the raw acceleration signals and the actual gravitational acceleration components obtained from a six-degree-of-freedom (6-DOF) flight dynamics simulator. Through this mapping, the model can identify the direction of gravity even during highly dynamic maneuvers or in the presence of measurement bias and noise. This network thus provides continuous attitude information throughout the entire flight envelope, regardless of GNSS signal availability.

The second network is trained to estimate the double-difference carrier-phase ambiguities between pairs of GNSS antennas and satellites. Using synthetic data produced by a GNSS signal generator, the model learns to recover the ambiguity term $\nabla N_{a_{i},a_{n}}^{S_{j},S_{m}}$ from measurable quantities such as corrected pseudo-range double differences and residual carrier-phase errors. By resolving these ambiguities, the network supports the angular restitution process described before, providing high-precision attitude information when satellite signals are available.

In combination, these two neural estimators form the backbone of the proposed hybrid learning-based navigation framework. The gravity network ensures robust, self-contained attitude estimation during all flight conditions, while the GNSS ambiguity network enhances accuracy and long-term stability when external signals are accessible. Together, they create a redundant and synergistic system capable of maintaining navigation performance even under partial sensor degradation.

The remainder of this section describes the adopted machine learning workflow for both networks, including data generation and preprocessing, model architecture and hyperparameter selection, training and validation methodology, error definition and evaluation metrics, and the experimental setup used to assess performance. By integrating physics-informed simulation data with supervised neural learning, the approach bridges model-based flight dynamics and data-driven inference, offering a scalable, accurate, and resilient alternative to traditional inertial navigation solutions.

#### 3.2.1. Machine Learning Architectures

Once the datasets have been defined and introduced, the architectures adopted for both neural models are described below. Both problems share a nonlinear nature and rely on sensor data contaminated by bias and measurement noise, making neural networks (NNs) a natural choice due to their universal approximation capability and robustness to imperfect input data [47,48,49].

(1) **Gravity Vector Estimation Network.** The first network is devoted to the reconstruction of the gravity vector in the body reference frame from accelerometer measurements. Its inputs correspond to the noisy accelerations $a_{m}={\left[a_{x_{b}},\;a_{y_{b}},\;a_{z_{b}}\right]}^{T}$, while the outputs are the estimated components of the local gravity vector ${\tilde{g}}_{b}={\left[{\tilde{g}}_{x_{b}},\;{\tilde{g}}_{y_{b}},\;{\tilde{g}}_{z_{b}}\right]}^{T}$, as defined in Equation (37). This network learns the nonlinear mapping between instantaneous accelerations and gravitational direction obtained from 6-DOF flight simulations, thus compensating for motion-induced effects. A feedforward multilayer perceptron (MLP) is used as the baseline model, augmented with dropout regularization to avoid overfitting. Since the relationship between inputs and outputs is instantaneous, no temporal recurrence is required in this configuration.
(2) **GNSS Phase Ambiguity Estimation Network.** The second network estimates the double differences of carrier phase ambiguity $\nabla N_{i,n}^{j,m}$ between pairs of GNSS sensors and satellites. Each network takes as input the actual measured double-difference carrier-phase and code observations, which inherently consist of the ideal values plus their corresponding measurement error terms, $\nabla \rho_{i,n}^{j,m}-\nabla E_{i,n}^{j,m}$, and outputs the corresponding ambiguity difference $\nabla N_{i,n}^{j,m}$. For notation simplicity, satellite indices $\left[S_{j},\ldots ,S_{m}\right]$ are written as [j,…,m] and sensor indices $\left[a_{i},\ldots ,a_{n}\right]$ as [i,…,n]. A dedicated neural network is trained for each $\nabla N_{i,n}^{j,m}$, ensuring specialization and avoiding cross-dependence among outputs. Because consecutive observations are time-correlated, a sliding time window of size *k* is applied to include temporal dependencies. Thus, for 36 inputs and a window size of *k*, each prediction is generated from 36×k features (the current and previous k−1 time steps). The simplest case, k=1, corresponds to the MLP baseline, while larger windows are processed through recurrent architectures.
(3) **Recurrent Network Structure.** To exploit the temporal continuity of the GNSS signals, Recurrent Neural Networks (RNNs) are employed. Each network instance *R* processes a sequence of inputs $in_{m}^{n}$ from simulation *n* at time step *m*, together with its previous hidden state $s_{m}^{n}$, to generate an output $out_{m}^{n}$ and an updated hidden state $s_{m+1}^{n}$. At the beginning of each simulation, all hidden states $s_{1}^{i}$ are initialized to zero.
  Among recurrent architectures, the Long Short-Term Memory (LSTM) network [50] is particularly relevant [51,52], as it addresses the short-term memory limitation present in basic RNNs. However, since the GNSS ambiguity prediction problem is mainly influenced by recent measurements, simpler recurrent layers (SimpleRNN) can also achieve satisfactory performance. The remaining layers are fully connected (Dense), and dropout regularization is applied before each dense layer to prevent overfitting in both MLP and RNN configurations.
  Both neural network systems—the gravity estimation model and the GNSS ambiguity estimation model— share the same training principles, optimization strategy, and evaluation methodology described in Section 3.2.4. Together, they enable the determination of the vehicle’s attitude through two complementary pathways: (1) indirect estimation via gravity vector reconstruction from accelerometers, and (2) direct estimation via angular restitution from GNSS phase measurements. The fusion of both networks’ outputs results in a unified and redundant attitude determination framework robust to sensor degradation, noise, and partial signal loss.

#### 3.2.2. Pre/Post-Processing

Prior to training, all datasets are preprocessed to ensure numerical stability and consistent learning behavior across both neural network architectures. All input variables are normalized using a min–max scaling procedure [53], which maps each feature into the range [0,1] based on the minimum and maximum values computed from the training set.

For supervised learning, each input vector must be paired with its corresponding target output. The specific preprocessing and post-processing steps depend on the type of network and its target variable.

##### Gravity Vector Estimation Network

The three accelerometer measurements are normalized using the global min–max scaling described above. The gravity components are scaled linearly to the range [0,1] during training, and the network outputs are mapped back to physical units after inference using the inverse transformation

(38) $${\tilde{g}}^{b}=g_{\min}^{b}+o⊙\left(g_{\max}^{b}-g_{\min}^{b}\right),$$

where o denotes the normalized network output vector and ⊙ indicates element-wise multiplication.

##### GNSS Ambiguity Estimation Network

The target variable for this model is the double-difference carrier-phase ambiguity $\nabla N_{i,n}^{j,m}$, which takes integer values in a bounded range, typically $\nabla N_{i,n}^{j,m}\in \left[-16,16\right]$. The inputs, consisting of double-differenced pseudo-range and error-corrected carrier-phase terms, are normalized to [0,1] using the same min–max procedure as for the gravity model.

In the classification configuration, $\nabla N_{i,n}^{j,m}$ is encoded with a standard one-hot vector over all admissible integer values, and the predicted ambiguity is obtained during inference by taking the index of the largest output component (argmax). In the regression configuration, $\nabla N_{i,n}^{j,m}$ is linearly mapped to [0,1] for training and rescaled back to its physical integer range after inference.

#### 3.2.3. Error Definition and Success Criteria

The evaluation of the two proposed neural network architectures— the gravity vector estimation network and the GNSS carrier-phase ambiguity network— is based on a unified set of quantitative metrics designed to assess prediction accuracy, consistency, and robustness under different operational conditions.

Each model is composed of a collection of neural networks that share the same overall architecture and hyperparameter configuration but are trained to estimate distinct target variables. For the gravity estimation network, each instance learns to predict one of the components of the local gravity vector ${\tilde{g}}_{b}={\left[{\tilde{g}}_{x_{b}},{\tilde{g}}_{y_{b}},{\tilde{g}}_{z_{b}}\right]}^{T}$ from accelerometer measurements. For the GNSS ambiguity network, each network within the ensemble is trained to infer a particular double-difference carrier-phase ambiguity $\nabla N_{i,n}^{j,m}$, corresponding to a specific combination of sensors $\left(a_{i},a_{n}\right)$ and satellites $\left(S_{j},S_{m}\right)$. Although the predicted variables differ in physical nature, both network families share a common learning structure and validation methodology.

Because the networks trained for different sensor-satellite combinations exhibit analogous behavior and statistical distributions, a representative subset of networks is used for comparison and analysis. For the GNSS-based model, one reference network corresponding to j=0, m=1, i=0, and n=1 is selected as the benchmark for performance evaluation. Similarly, for the gravity estimation model, a representative configuration corresponding to a nominal 6-DOF flight condition is used to compute global accuracy and error metrics.

The following performance indicators are used to quantify model quality. Model performance is assessed using accuracy and mean squared error (MSE). Accuracy is reported for the classification-based ambiguity networks, whereas MSE is used for both gravity and ambiguity estimation. For the GNSS ambiguity estimator, MSE is computed only over failed predictions, since small integer deviations in $\nabla N_{i,n}^{j,m}$ can produce large errors in the reconstructed attitude and position at the GNSS wavelength scale.

To enable direct comparison between tasks with different physical units, we also report a normalized mean squared error defined as

(39) $${\mathrm{MSE}}_{\mathrm{norm}}=\frac{1}{N}\sum_{k=1}^{N}{\left(\frac{{\hat{y}}_{k}-y_{k}}{\max(y)-\min(y)}\right)}^{2},$$

which yields a dimensionless metric common to both networks. In the results, ${\mathrm{MSE}}_{\mathrm{norm}}$ and its square root (normalized RMSE) are used interchangeably.

In general, classification networks tend to achieve higher prediction accuracy, while regression-based architectures yield smaller normalized mean squared errors for incorrect predictions. This behavior reflects the different optimization objectives used during training— categorical cross-entropy loss for classification and mean-squared error minimization for regression. Nevertheless, when classification accuracy approaches 100%, isolated misclassifications may lead to comparatively large normalized MSE values. Conversely, regression networks may show smoother performance degradation at the cost of slightly lower overall accuracy.

The same evaluation framework is consistently applied to both neural network families, ensuring a fair comparison of their predictive capabilities and highlighting their complementary roles within the hybrid learning-based navigation system.

**Loss functions and training objectives.** From an optimization viewpoint, both neural estimators are trained by minimizing standard supervised learning losses built from the simulated datasets.

For the GNSS ambiguity estimation problem, a dedicated neural network is trained for each double-difference carrier-phase ambiguity term $\nabla N_{i,n}^{j,m}$ between sensors $a_{i},a_{n}$ and satellites $S_{j},S_{m}$. For clarity, the compact notation introduced in Section 3.2.1 is retained. Let $z_{k}^{N}$ denote the input feature vector (containing corrected pseudo-range double differences, residual carrier-phase errors, and geometric quantities) for sample *k*, and let $\nabla N_{i,n,k}^{j,m}$ be the corresponding ground-truth ambiguity value.

In the *classification* formulation, the network outputs a discrete probability distribution:

(40) $$p_{\theta }\;\left(\nabla N_{i,n}^{j,m}|z_{k}^{N}\right)$$

and the trainable parameters θ are obtained by minimizing the empirical categorical cross-entropy:

(41) $$J_{\mathrm{CE}}\left(\theta \right)=-\frac{1}{K}\sum_{k=1}^{K}\log p_{\theta }\;\left(\nabla N_{i,n,k}^{j,m}|z_{k}^{N}\right)$$

In the *regression* formulation, the network predicts a real-valued estimate ${\hat{\nabla N}}_{i,n,k}^{j,m}={\hat{\nabla N}}_{i,n}^{j,m}\left(z_{k}^{N};\theta \right)$, and training is performed by minimizing the mean squared error:

(42) $$J_{\mathrm{MSE}}\left(\theta \right)=\frac{1}{K}\sum_{k=1}^{K}{\left(\nabla N_{i,n,k}^{j,m}-{\hat{\nabla N}}_{i,n}^{j,m}\left(z_{k}^{N};\theta \right)\right)}^{2}$$

The gravity vector estimation model is also trained as a regression problem. Given input accelerometer measurements in body axes $a_{k}^{m}$ and the corresponding ground-truth gravity vectors $g_{k}^{b}={\left[g_{x,k}^{b},g_{y,k}^{b},g_{z,k}^{b}\right]}^{⊤}$, the network output ${\hat{g}}_{k}^{b}={\hat{g}}^{b}\left(a_{k}^{m};\theta_{g}\right)$ is optimized by minimizing:

(43) $$J_{g}\left(\theta_{g}\right)=\frac{1}{N_{g}}\sum_{k=1}^{N_{g}}{∥g_{k}^{b}-{\hat{g}}^{b}\left(a_{k}^{m};\theta_{g}\right)∥}_{2}^{2}$$

In all cases, the Adam optimizer is used with the hyperparameters and training settings described in Section 3.2.4, ensuring a consistent workflow for both the ambiguity and gravity estimation networks.

#### 3.2.4. Experimental Methodology

This section describes the methodology used to train, tune, and validate both neural models.

All experiments were conducted using the *TensorFlow v2.16* framework with the *Keras* API. The dataset was divided into three disjoint subsets, allocating 70% of the data for training, 15% for validation, and the remaining 15% for testing. Both models were trained under a common baseline hyperparameter configuration. Each network consisted of five dense layers with 800, 400, 200, 100, and an output layer whose size was set to 33 for the classification task and 1 for the regression task. The *ReLU* activation function was applied to all intermediate layers, while the output layer employed a *softmax* activation for classification and a *sigmoid* activation for regression.

A recurrent layer with 125 neurons and *ReLU* activation was also included, with a dropout rate of 0.1 to prevent overfitting. The optimization process was carried out using the *Adam* optimizer with an initial learning rate of 0.001. Training was performed for 600 epochs, using a batch size of 200 in the case of the MLP networks and 16 for the RNN architectures. Finally, data were shuffled before each epoch.

To ensure consistency, all training procedures used early stopping or checkpointing mechanisms, preserving the best model based on validation performance. Once optimal configurations were identified, the validation data were merged with the training data for final model refinement.

##### Phase 1—Task and Architecture Selection

The first phase aims to determine the most appropriate learning paradigm and neural network architecture for each model. For the GNSS ambiguity estimation problem, the two main formulations—classification and regression—are tested using both a multilayer perceptron (MLP) and a recurrent neural network (RNN) with a simple recurrent layer (*SimpleRNN*). For the gravity estimation task, a regression formulation is employed exclusively, but both network types (MLP and RNN) are evaluated to compare temporal learning benefits.

Each configuration is trained three times with different random seeds and evaluated across three time-window sizes (k=1,5,10). The baseline configuration corresponds to an MLP with k=1. This phase thus comprises 24 total experiments. The most promising combination of task formulation (classification or regression) and network type (MLP or RNN) is selected for subsequent phases.

##### Phase 2—Architecture Optimization

Once the preferred task and network type are established, the second phase focuses on refining the internal architecture parameters. For both networks, several dense-layer configurations are explored: (800,400,200,100), (400,200,100,50), and (200,100,50,25) neurons per layer. To maintain computational tractability, the total number of dense layers is limited to five, including input and output layers.

For recurrent models, the number of neurons in the recurrent layer is varied across {100,125,150,175,200}, and two activation functions (*ReLU* and *tanh*) are compared. Each configuration is executed three times to ensure repeatability and statistical reliability. The optimal architecture from this phase— defined as the one achieving the highest accuracy and/or lowest MSE on the validation set— is used as the foundation for the following phase.

##### Phase 3—Learning Rate and Epoch Tuning

The third phase focuses on optimizing training duration and learning rate parameters for the selected architecture. Five different initial learning rates are tested: 0.01, 0.005, 0.001, 0.0005, and 0.0001. Each configuration is trained for up to 2000 epochs, allowing the network to reach convergence or early stopping based on validation loss. As in previous phases, three repetitions per configuration are performed using different random seeds to ensure robustness of the results. This phase determines the most effective trade-off between convergence speed and final accuracy.

##### Phase 4—Final Model Training and Integration

In the final phase, the best architecture and hyperparameter settings identified in the earlier stages are used to train the complete models. The validation set is merged with the training data, yielding an 80–20% split between training and testing subsets. This enlarged training set improves generalization and reduces overfitting risks, even without checkpointing. Each $\nabla N_{i,n}^{j,m}$ value in the GNSS ambiguity model is estimated by an independent neural network within the ensemble, while the gravity estimation model produces three simultaneous outputs corresponding to the components of $g_{b}$.

A reliability threshold is established for model inclusion: 99% minimum accuracy for classification networks and an MSE<0.9 for regression networks. Networks that do not meet this criterion are retrained using identical configurations but different random seeds until they reach the desired performance. The training process concludes once all networks in both models satisfy these reliability standards, ensuring a consistent and high-fidelity hybrid learning-based navigation system.

## 4. Results, Discussion, and Selection of the Final ML Model

The experimental campaign was organized into five consecutive phases, each building on the outcomes of the previous one:

1 Determination of the optimal neural network type and learning task (regression or classification).
2 Identification of the most suitable network architecture and parameter configuration.
3 Fine-tuning of the initial learning rate and number of epochs.
4 Generation of the final model, where each output is predicted by an individual network.
5 Evaluation of the model’s performance when applied to an angular restitution problem.

The following sections present the main results obtained at each stage and the rationale for selecting the optimal configuration for both neural network families.

### 4.1. Phase 1—Selection of the Neural Network Type and Learning Task

Training deep neural models can occasionally lead to convergence to local minima. To mitigate this issue and ensure the reliability of the results, each configuration was trained three times with different random seeds, and the best-performing iteration was retained for comparison.

**Figure 3:**
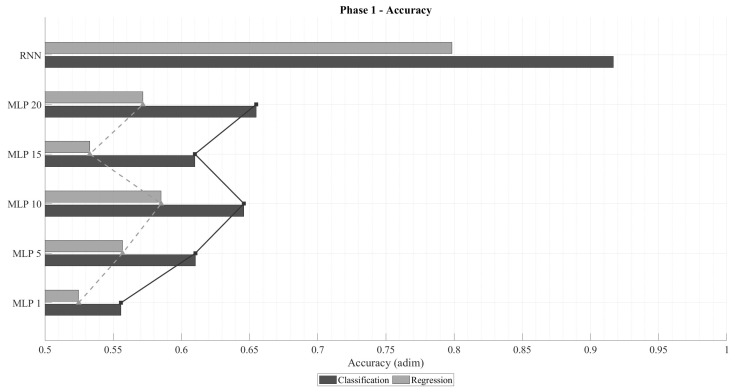
Accuracy of MLP and RNN architectures in classification and regression tasks for different time-window lengths.

Figure 3 illustrates the accuracy obtained for both regression and classification tasks using Multi-Layer Perceptrons (MLP) with various sliding time-window lengths, as well as Recurrent Neural Networks (RNN). As expected, classification networks outperform regression ones in terms of accuracy, since their training objective explicitly minimizes categorical cross-entropy. A clear trend can be observed: accuracy improves as the time window increases, up to an optimal range beyond which performance begins to degrade. For regression, the optimal window appears near k=5, whereas for classification, the maximum likely occurs between k=20 and k=30. This pattern aligns with physical intuition: the relevance of past samples diminishes over time, and excessively long input sequences increase the model’s complexity, leading to reduced generalization capability. In all cases, RNN architectures consistently outperform their MLP counterparts, confirming that temporal dependencies play a critical role in predicting $\nabla N_{i,n}^{j,m}$.

**Figure 4:**
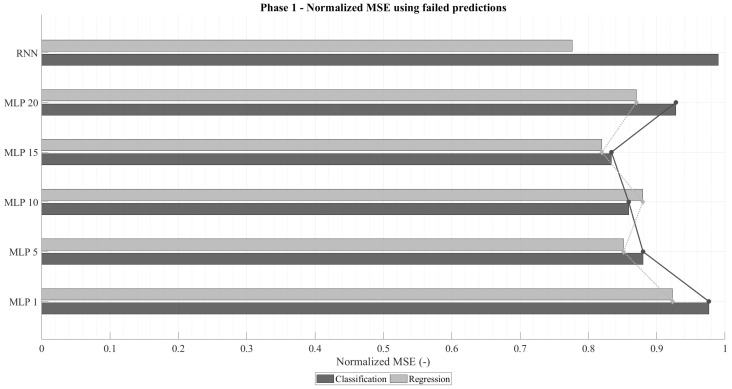
Normalized MSE in failed predictions for MLP and RNN architectures under regression and classification tasks.

Figure 4 presents the normalized mean squared error ($MSE_{norm}$) computed only for failed predictions. As anticipated, regression networks exhibit lower errors than classification ones, given that their loss function directly minimizes MSE. Analogously to the accuracy trend, $MSE_{norm}$ decreases with increasing time-window size, reaching a minimum before rising again. In an ideal failure scenario, the error would correspond to a one-unit deviation from the correct value (i.e., $MSE_{norm}=1$). Among them, the RNN-regression models consistently achieved the lowest $MSE_{norm}$ values, while RNN-classification networks produced the highest. This is attributable to the fact that classification networks prioritize categorical accuracy, occasionally making riskier predictions that result in larger deviations when they fail.

**Figure 5:**
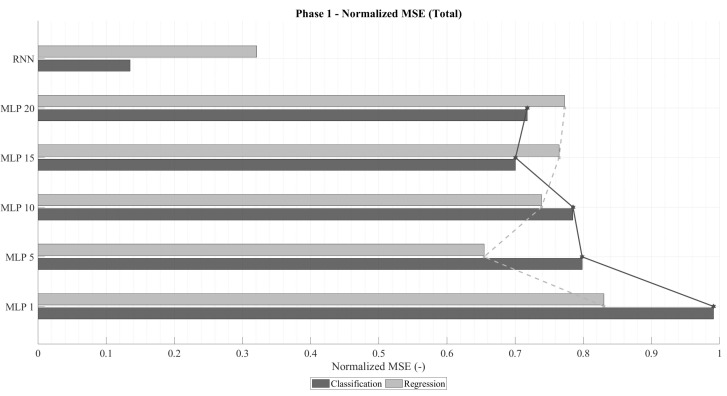
Total normalized MSE for MLP and RNN architectures in classification and regression configurations.

The emphasis on analyzing the $MSE_{norm}$ in the case of failure stems from the fact that highly accurate classification networks naturally exhibit low overall MSE, even when their individual mispredictions deviate substantially. This effect is illustrated in Figure 5, which shows the total normalized MSE across all predictions. Despite regression models being trained to minimize this metric, classification networks can outperform them in total $MSE_{norm}$ whenever their accuracy becomes sufficiently high. This trend becomes particularly evident for longer time windows in both MLP and RNN configurations.

In summary, two architectures stand out from this phase: (1) a recurrent classification network with very high accuracy and moderate deviations in failed predictions, and (2) a recurrent regression network with lower accuracy but notably smaller deviations when errors occur. These two configurations will serve as the basis for the subsequent analysis, where architectural refinements and parameter optimizations are explored to further enhance model performance.

### 4.2. Phase 2—Selection of the Architecture and Parameters

This phase focuses on identifying the most suitable neural network architecture and parameter configuration for both learning tasks. Three network scales were tested to assess the influence of architectural complexity on performance:

- **Big Architecture (BA)**: 800, 400, 200, and 100 neurons in the dense layers.
- **Medium Architecture (MA)**: 400, 200, 100, and 50 neurons.
- **Little Architecture (LA)**: 200, 100, 50, and 25 neurons.

Within each architecture, several recurrent configurations were examined, varying the number of neurons in the recurrent layer (100, 125, 150, 175, and 200) and the activation function (*tanh* and *ReLU*). As in Phase 1, each configuration was trained three times with different random seeds, and the best-performing repetition was selected for evaluation.

The evaluation metrics were chosen according to the learning formulation: accuracy for classification networks and normalized MSE ($MSE_{norm}$) for regression networks, considering only failed predictions. This normalization enables a consistent comparison between architectures of different sizes and output ranges.

**Figure 6:**
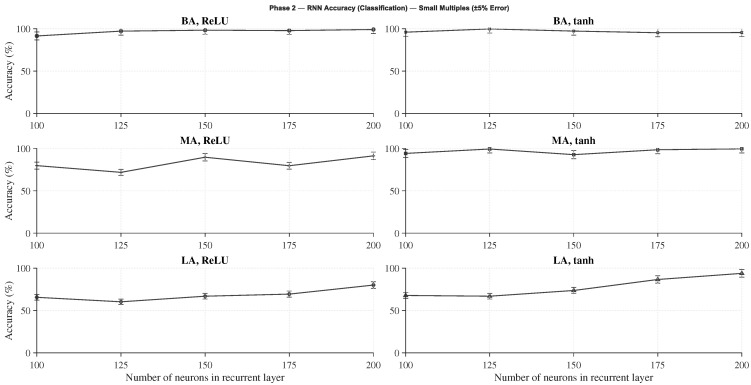
Accuracy of classification networks with different RNN architectures and activation functions.

Figure 6 shows the classification accuracy obtained across the various architectures and configurations. The results reveal that using the *tanh* activation function in the recurrent layer consistently outperforms *ReLU*. Moreover, larger architectures achieve higher accuracy, though the improvement between the Medium (MA) and Big (BA) configurations is relatively modest. The Big Architecture using *tanh* shows stable accuracy across all recurrent-layer sizes (from 100 to 200 neurons), suggesting robustness with respect to this parameter. Therefore, to balance performance and computational efficiency, the configuration with 100 recurrent neurons is selected as the reference model for the classification task.

**Figure 7:**
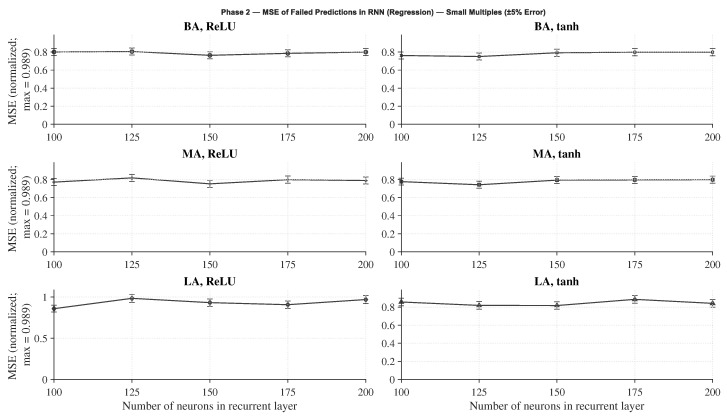
Normalized MSE in failed predictions for regression networks using different RNN architectures and activation functions.

**Figure 8:**
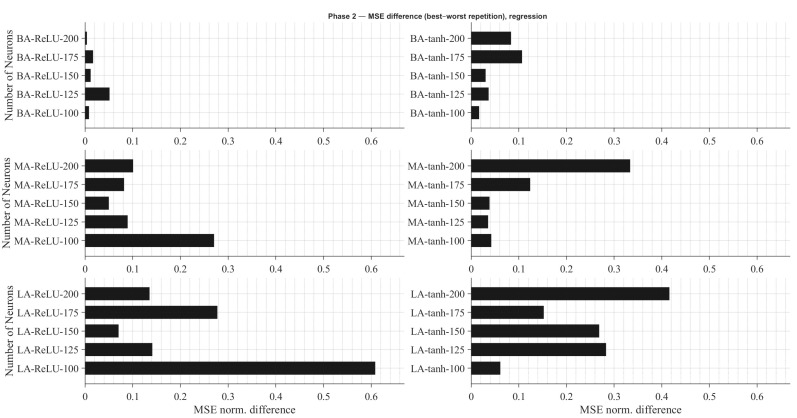
Difference in normalized MSE between best and worst training repetitions for each RNN architecture in regression tasks.

Figure 7 presents the normalized mean squared error ($MSE_{norm}$) for the regression networks under the same architectural variations. Similar to the classification case, larger architectures and the use of *tanh* activation yield the best results, with optimal performance observed at 100 recurrent neurons. The performance gap between Medium and Big Architectures is minimal, indicating that the MA configuration could serve as a computationally lighter alternative with negligible accuracy loss. However, when considering robustness to initialization, the Big Architecture shows smaller sensitivity to seed variability, as depicted in Figure 8.

This figure quantifies the difference in $MSE_{norm}$ between the best and worst training repetitions for each tested architecture. It is evident that variability decreases as the network size increases, confirming that larger models provide more stable convergence behavior. Consequently, the Big Architecture with 100 recurrent neurons and *tanh* activation in the recurrent layer is selected as the preferred configuration for regression networks as well.

In summary, both analyses indicate that the Big Architecture (BA) with 100 recurrent neurons and *tanh* activation provides the most balanced trade-off between accuracy, stability, and computational cost for both network types. The next phase focuses on fine-tuning the learning rate and number of epochs to further refine the performance of this selected configuration.

### 4.3. Phase 3—Setting the Number of Epochs and Initial Learning Rate

This phase aims to determine the optimal initial learning rate (LR) and number of training epochs required to obtain a well-converged model before proceeding to the final training phase. In Phase 4, the selected configuration will be trained using an expanded dataset, without checkpoints or a dedicated validation set, in order to maximize the amount of data available for learning.

A total of 2000 epochs were executed for each experiment, testing five different initial learning rates: 0.01, 0.005, 0.001, 0.0005, and 0.0001. For each learning rate, the best-performing repetition—defined as the one achieving the highest accuracy on the test set for classification networks—was selected.

**Figure 9:**
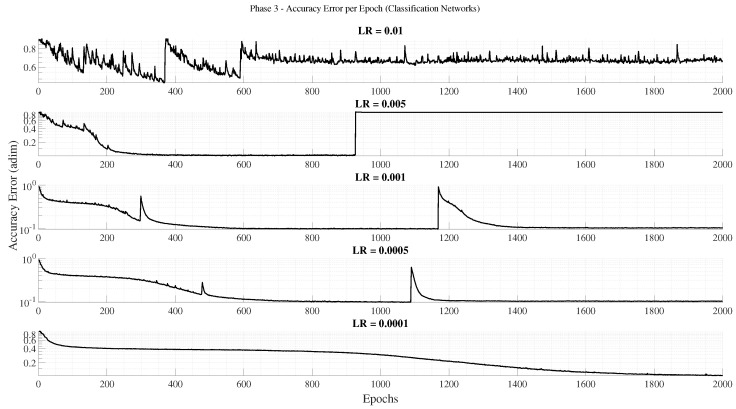
Accuracy error (1−accuracy) on the validation set across epochs for different initial learning rates in classification networks.

Figure 9 summarizes the validation accuracy error across epochs for the different initial learning rates. Intermediate values (0.005, 0.001, 0.0005) provide better generalization than the extreme cases, and the lowest validation error is obtained with an initial learning rate of 0.0005. Consequently, this learning rate was adopted for the subsequent phases, together with a reduced maximum training horizon of 600 epochs for the final models.

For regression networks, the training dynamics differ from those of classification. While these models can achieve low global mean squared error ($MSE_{norm}$), they may still yield relatively large deviations in failed predictions. Beyond a certain number of epochs, the network tends to prioritize minimizing overall $MSE_{norm}$ by tolerating a few large errors rather than many small ones— a behavior consistent with the trends observed in Figure 6. To prevent this imbalance and maintain consistent accuracy across predictions, the regression models in Phase 4 will adopt an initial learning rate of 0.001 and a total of 600 training epochs, representing a compromise between convergence quality and computational efficiency.

In summary, the classification networks perform best with an initial learning rate of 0.0005 and approximately 1050 epochs, whereas the regression networks achieve their optimal trade-off at a learning rate of 0.001 and 600 epochs. These values will serve as the baseline configuration for the final model training in the next phase.

### 4.4. Phase 4—Training the Final Model

In this final phase, the complete models were trained and validated—one neural network for each $\nabla N_{i,n}^{j,m}$ value—using the optimal architecture and parameters determined in the previous stages. To maximize the available data, the validation set was merged with the training set, thereby enlarging the effective training base. Unlike the earlier phases, no checkpoints were used; instead, the final network obtained at the end of training was retained for evaluation. If a network failed to meet the reliability thresholds—defined as at least 99% accuracy for classification networks or a normalized mean squared error ($MSE_{norm}$) lower than 1.1 in the case of regression networks—training was restarted from scratch with a different random seed until the desired criteria were achieved.

**Figure 10:**
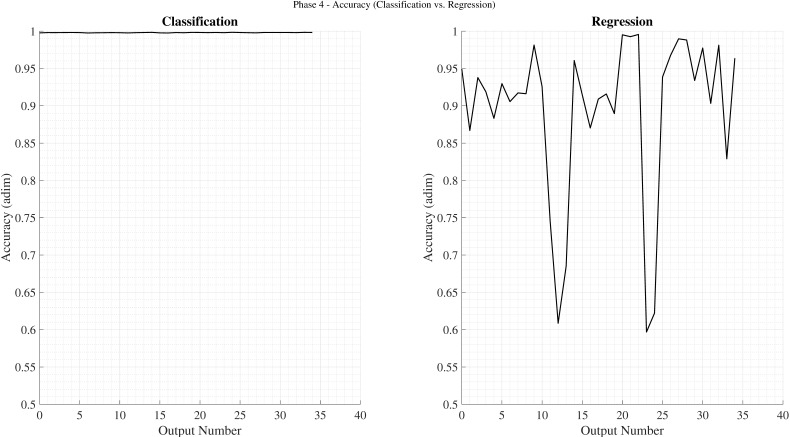
Accuracy of the final classification and regression networks.

**Figure 11:**
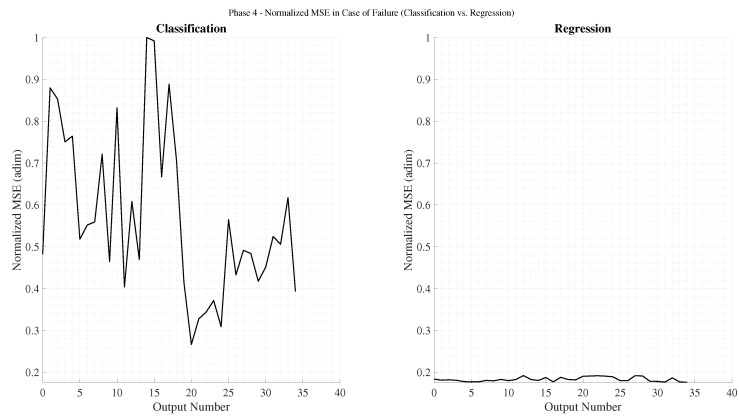
Normalized MSE in failed predictions for the final classification and regression networks.

Figure 10 presents the accuracy obtained by the final classification and regression networks. Although the regression networks display greater variability, none of them surpass the classification networks in accuracy. A similar trend is observed when analyzing $MSE_{norm}$ values for failed predictions, shown in Figure 11. These results confirm that minimizing the frequency of failures with larger deviations (as achieved by classification networks) is preferable to minimizing smaller errors that occur more frequently (as seen in regression models). Notably, the lowest $MSE_{norm}$ achieved by the regression models is only marginally higher than that of the best-performing classification model.

#### Temporal Dependence of Prediction Failures

A deeper analysis was conducted to determine whether prediction failures are temporally correlated—that is, whether an error at time step *t* is influenced by an error at time step t−1. To evaluate this, results from 1000 independent simulations were analyzed. Each simulation recorded the number of prediction failures per historical data sequence (the test set includes 20 historical datasets of 5760 samples each) across all 35 networks comprising the model. Synthetic results were then generated by randomly redistributing the same number of failures uniformly across time. The number and size of failure clusters were then tallied and averaged across the 1000 trials.

**Figure 12:**
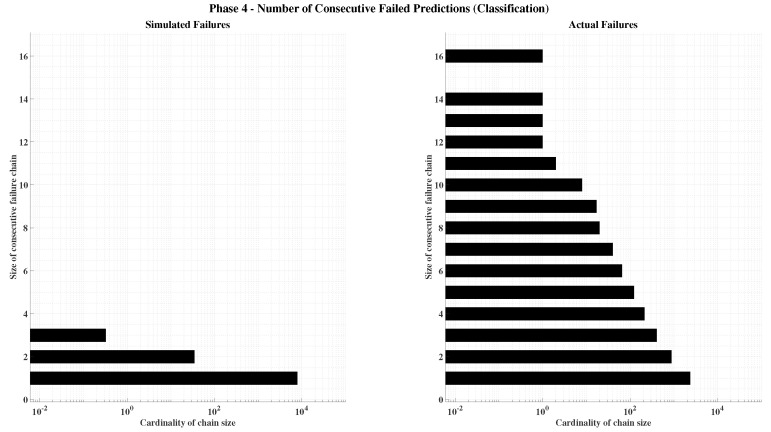
Cardinality of consecutive failures in the final classification model.

**Figure 13:**
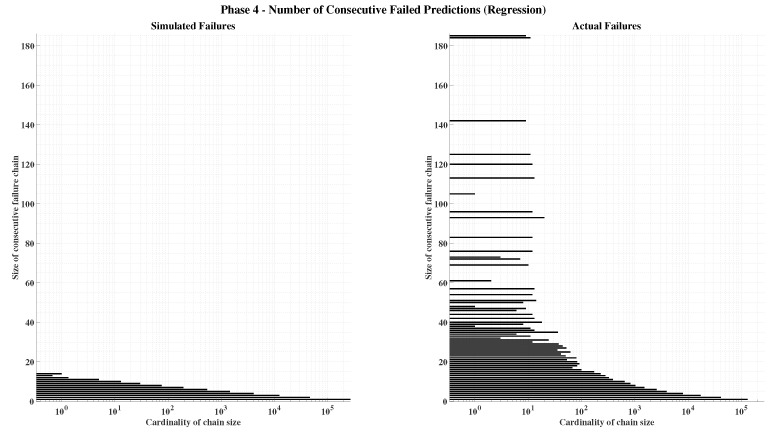
Cardinality of consecutive failures in the final regression model.

As shown in Figure 12, the simulated classification data display a greater number of isolated failures (single events uncorrelated with neighboring time steps) compared with the real model outcomes. However, the actual model produces larger and more diverse failure clusters—up to 16 consecutive time steps— whereas the randomized data rarely exceed four. A similar yet more pronounced behavior appears in the regression models (Figure 13), reflecting their lower overall accuracy. Empirically, this confirms that the conditional probability of failure given a previous failure is higher than the probability of failure given a previous success, i.e., P(failureatt∣failureatt−1)>P(failureatt∣successatt−1). This observation is consistent with the temporal nature of RNNs, where consecutive inputs are similar and thus yield correlated prediction outcomes. It should be noted, however, that in a Simple RNN architecture, the recurrent connections propagate processed input information— not previous prediction errors—between consecutive time steps.

#### Inter-Network Dependence of Prediction Failures

**Figure 14:**
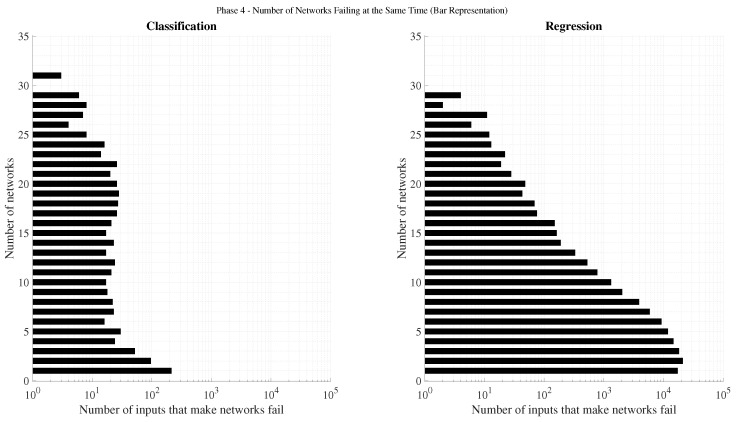
Cardinality of simultaneous incorrect outputs across the 35 networks in the final classification and regression models.

Each input sample is processed by all 35 networks within the model, resulting in 35 parallel outputs per time step. To assess whether failures in one network influence those in others, the number of simultaneous incorrect outputs across networks was analyzed. Figure 14 shows the distribution of instances in which *K* networks (0≤K≤34) failed simultaneously for the same input.

In the worst-case scenario, assuming a 1% individual failure rate (99% accuracy), the probability of 20 networks failing concurrently for the same input would be on the order of $0.01^{20}$, or roughly one event every $10^{40}$ samples. Nevertheless, this phenomenon was observed more than ten times within only 115,200 instances. This clearly indicates statistical dependence between networks: the probability that a network *m* fails given that another network *n* also fails at the same time step is significantly higher than the probability of failure given that the remaining networks succeed, i.e.,

$$\begin{array}{cc} P(\mathrm{failure}\;\mathrm{of}\;\mathrm{network}\;m\;\mathrm{at}\;t|\mathrm{failure}\;\mathrm{of}\;\mathrm{network}\;n\;\mathrm{at}\;t) & >\\ P(\mathrm{failure}\;\mathrm{of}\;\mathrm{network}\;m\;\mathrm{at}\;t|\mathrm{success}\;\mathrm{of}\;\mathrm{networks}\;p\;\mathrm{at}\;t) \end{array}$$

where m,n∈[0,34], m≠n, and p∪m=[0,34].

This interdependence arises from the inherent architectural and parameter similarities among the networks, which were trained independently but share the same structural design and data characteristics. As a result, they exhibit correlated behavior under certain input conditions, particularly when the input features lie near the network decision boundaries.

The following subsection presents the angular restitution results, demonstrating the applicability and performance of the proposed algorithms in a complete attitude determination scenario.

### 4.5. Angular Restitution Results

In order to assess the effectiveness of the proposed attitude determination algorithm, a non-linear flight dynamics model, as developed by [54], was employed in a series of simulation experiments. Monte Carlo simulations were used to evaluate the performance of the closed-loop system [54] under various sources of uncertainty, including initial conditions, sensor data acquisition, atmospheric conditions, thrust characteristics, and aerodynamic coefficients (with their associated dispersion bounds).

**Vector pairs used for restitution.** Consistently with the methodology established in the previous sections, the angular restitution relies on pairs of vectors defined in both the Earth-fixed and body-fixed frames to reconstruct the rotation matrix (see Equation (20)). In this work, two complementary sources of vector pairs were employed: (i) *Across the whole trajectory*, vector pairs derived from the outputs of the neural networks: the GNSS-based ambiguity network provides the inter-antenna baselines in the Earth frame (via the double-difference formulation previously defined, while the gravity-estimation network supplies the gravity direction in the body frame; these vectors are stacked to form the correspondence sets used in Equation (20). (ii) *During the terminal phase*, the pairs of vectors defined previously from the semi-active laser (SAL) seeker are incorporated, i.e., the line of sight (LOS) to the target in Earth axes from GNSS/geometry and the LOS in body axes from the SAL photodiode measurements. Thus, when SAL data becomes available, the restitution set is augmented with these SAL-based pairs, increasing observability and improving conditioning of the least-squares attitude solution.

The simulation tests involved the execution of a predefined set of nominal, regular Euler-angle commands to enable a direct comparison between the estimated and true attitudes. Two families of commands were used: (a) an ascending ramp with constant slope between 0 and 10 s, reaching final amplitudes from 5° to 45° in 5° steps; and (b) a sinusoid at 1 rad/s with amplitudes in the same range. All runs were carried out in MATLAB/Simulink R2024b on a desktop computer (2.8 GHz CPU, 8 GB RAM).

To quantify restitution quality, the root mean squared error (RMSE) over the full simulation horizon is computed for each Euler angle as

(44) $$\begin{array}{ccccc} RMSE_{yaw} & =\sqrt{\frac{\oint {\left(\phi_{real}-\phi \right)}^{2}\;d\sigma }{\oint d\sigma }}, & \; & RMSE_{pitch} & =\sqrt{\frac{\oint {\left(\theta_{real}-\theta \right)}^{2}\;d\sigma }{\oint d\sigma }},\\ RMSE_{roll} & =\sqrt{\frac{\oint {\left(\psi_{real}-\psi \right)}^{2}\;d\sigma }{\oint d\sigma }}. \end{array}$$

As a reference baseline for the ambiguity-resolution approach using neural networks, a control simulation with RTK was executed under identical initial and boundary conditions. Signals were generated with an Orolia GSG-8 (Skydel engine) configured for RTK differential simulation [45]. Antenna positions, the vehicle reference point, and the motion profile were assumed known.

Results for regression-based networks (REG), classification-based networks (CLAS), and the RTK control are summarized in Table 5, Table 6 and Table 7. For each Euler angle, rows report RMSE for the ramp (“Constant”), the sinusoid (“Sin”), and their average. The last column gives the across-amplitude average. As anticipated, classification networks deliver RMSEs comparable to the classical RTK baseline and consistently lower than those of regression networks, while benefiting from the additional SAL-based vector pairs in the terminal segment.

These outcomes reflect the high redundancy and precision of the sensor suite: most samples are highly accurate, and the restitution least-squares fit (Equation (20)) effectively attenuates residual inconsistencies with largest MSE. For the angular restitution trials we adopted the “Big Architecture” defaults from Section 3.2.4 and trained for 2000 epochs to ensure convergence under the combined (NN+SAL) vector-set regime.

Overall, the average error across all amplitudes remains below one degree, which is fully compatible with guidance, navigation, and control requirements for low-cost aerial platforms. The joint use of NN-derived vector pairs throughout the trajectory and SAL-based pairs in the terminal segment proves effective and robust for attitude restitution across diverse operational scenarios.

## 5. Conclusions

This work presented a hybrid, learning-based navigation framework in which two neural networks estimate the gravity vector in body axes and the GNSS double-difference carrier-phase ambiguities ∇N, complemented in the terminal phase by a semi-active laser (SAL) seeker.

A multi-phase experimental campaign was carried out to (i) select the learning formulation and network family for each task (classification vs. regression, MLP vs. RNN), (ii) tune architectures, input time windows and other hyperparameters, (iii) adjust the learning rate and training horizon, and (iv) train the gravity vector estimator together with one specialized network per ambiguity term $\nabla N_{i,n}^{j,m}$ before validating the full angular restitution pipeline. These steps rely on standard supervised learning methods rather than on a new training algorithm; the contribution of the study lies in how such methods are configured, embedded and assessed within a realistic 6-DoF navigation and guidance framework. Across the different phases, classification-based ambiguity networks achieved the highest prediction accuracy, whereas regression models yielded the lowest *per-failure* errors; to compare both families on a common basis, the mean squared error was normalized with respect to the dynamic range of each output. The final model set—comprising one gravity network and one ambiguity network per $\nabla N_{i,n}^{j,m}$—meets the prescribed reliability thresholds, with the classification configuration providing the most favorable compromise between accuracy and normalized MSE in end-to-end attitude restitution.

Against an RTK control setup, the proposed approach achieved comparable precision, remaining within the same order of magnitude while relying on lower-cost sensing and without explicit analytical ambiguity resolution. Monte Carlo trials with a non-linear 6-DoF flight model confirmed robust closed-loop behavior under uncertainty in initial conditions, sensing, atmosphere, thrust, and aerodynamics. In the terminal segment, introducing SAL-based vector pairs improved conditioning and reduced restitution error, yielding average angular RMSEs below one degree across the tested maneuver set—values compatible with guidance, navigation, and control requirements for cost-effective aerial platforms.

**Scope and limitations.** To bound training cost and demonstrate feasibility, data generation assumed a fixed site (URJC, Madrid) and a 24 h window, with representative satellite visibility and sensor noise models. These constraints do not cover all geodetic conditions or constellation geometries. Nevertheless, the methodology is readily extensible: future work will (i) generalize GNSS simulation across latitude/longitude/altitude and time, (ii) reduce sensor count via architecture sharing or multiple neural network based learning, (iii) fuse both networks with classical filters for consistency checks, and (iv) exploit SAL more deeply for terminal-phase refinement and contingency handling.

**Main takeaway.** Learning gravity in body axes from accelerations and learning $\nabla N_{i,n}^{j,m}$ from GNSS observables provide two independent, mutually reinforcing vector sources for attitude determination—supplemented by SAL near impact. This redundancy, coupled with normalized, task-agnostic evaluation, delivers accurate, scalable attitude estimation suitable for onboard implementation in low-cost aerospace vehicles, and establishes a solid foundation for broader, global deployments in future studies.

## Figures and Tables

**Table 1:** Mass and inertia parameters of the aerial vehicle (values adjusted within ±2%).

Parameter	Value
Initial mass	63.05 kg
Fuel mass	21.35 kg
Moment of inertia $I_{x0}$	0.188 kg m^2^
Moment of inertia $I_{y0}$	19.10 kg m^2^
Maximum thrust	29.68 kN

**Table 2:** Aerodynamic coefficients of the vehicle.

Mach Number	0.00	0.40	0.60	0.70	0.80	0.90	1.00
$C_{L_{\alpha }}$	8.09	8.02	8.52	8.44	8.96	9.10	8.75
$C_{D_{0}}$	0.273	0.254	0.242	0.236	0.229	0.226	0.417
$C_{L_{\alpha^{3}}}$	19.65	19.82	18.88	18.60	18.34	17.63	44.75
$C_{D_{\alpha^{2}}}$	10.83	10.69	11.23	11.02	11.53	11.68	15.26
$C_{Nq}$	51.40	54.00	58.40	59.90	64.50	68.85	72.45
$C_{mf}$	−0.59	−0.63	−0.69	−0.73	−0.74	−0.77	−0.79
$C_{M_{q}}$	−229.00	−231.90	−250.25	−258.85	−263.40	−281.00	−291.20
$C_{M_{\alpha }}$	−36.05	−35.25	−36.40	−36.05	−36.85	−36.40	−35.65
$C_{M_{\alpha^{3}}}$	−16.50	−17.92	−20.18	−21.55	−23.60	−18.75	15.10
$C_{mm}$	3.08	3.32	3.63	3.78	3.90	4.02	4.18
$C_{N\alpha_{w}}$	0.00	0.41	0.44	0.45	0.43	0.46	0.44
$C_{spin}$	−0.04	−0.04	−0.04	−0.03	−0.03	−0.03	−0.03

**Table 3:** Experimental calibration between measured and actual spot displacement.

$r_{quad}$	0.47	1.00	1.52	2.05	2.62	3.71	5.80
$r_{c}$	0.1	0.2	0.3	0.4	0.5	0.6	0.7

**Table 4:** Sample of input–output data pairs for gravity vector estimation (subset of 10,000 samples obtained from 6-DOF simulation).

Sample	Input: Measured Accelerations (m/s^2^)			Output: True Gravity Vector (m/s^2^)		
	$a_{x_{b}}$	$a_{y_{b}}$	$a_{z_{b}}$	$g_{x_{b}}$	$g_{y_{b}}$	$g_{z_{b}}$
1	3.018	−2.010	−6.805	3.213	6.842	−4.391
…						
1000	2.156	1.043	−4.734	−7.023	4.550	2.337
…						
2000	−3.091	2.027	−1.788	1.961	−9.440	0.988
…						
9000	5.362	−3.058	−1.610	−5.117	−7.019	−3.165
…						
10,000	−1.349	2.025	−3.846	−2.099	−3.153	−8.573

**Table 5:** Root Mean Squared Errors for roll angles.

	Roll (deg.)									
**Angle (deg)**	**5**	**10**	**15**	**20**	**25**	**30**	**35**	**40**	**45**	**Av. RMSE**
**Constant**										
RTK	0.28	0.33	0.42	0.57	0.67	0.62	0.78	0.72	0.93	0.59
CLAS	0.25	0.35	0.36	0.57	0.70	0.68	0.75	0.73	0.84	0.58
REG	2.63	5.21	3.69	7.92	8.54	8.98	7.97	9.29	9.12	7.04
**Sin**										
RTK	0.27	0.30	0.36	0.50	0.59	0.65	0.71	0.77	0.69	0.54
CLAS	0.26	0.34	0.37	0.44	0.60	0.62	0.66	0.70	0.74	0.53
REG	3.22	4.29	4.37	5.48	6.28	6.09	9.52	9.60	10.14	6.55
**Av. RMSE**										
RTK	0.28	0.32	0.39	0.54	0.63	0.64	0.75	0.75	0.81	0.56
CLAS	0.26	0.35	0.37	0.51	0.65	0.65	0.71	0.72	0.79	0.55
REG	2.93	4.75	4.03	6.70	7.41	7.54	8.75	9.45	9.63	6.80

**Table 6:** Root Mean Squared Errors for pitch angles.

	Pitch (deg.)									
**Angle (deg)**	**5**	**10**	**15**	**20**	**25**	**30**	**35**	**40**	**45**	**Av. RMSE**
**Constant**										
RTK	0.29	0.33	0.39	0.41	0.50	0.54	0.57	0.59	0.76	0.49
CLAS	0.26	0.34	0.38	0.48	0.53	0.50	0.61	0.68	0.70	0.50
REG	4.23	3.81	4.50	5.81	5.46	5.99	6.82	6.93	8.08	5.74
**Sin**										
RTK	0.26	0.35	0.37	0.44	0.47	0.50	0.53	0.54	0.61	0.45
CLAS	0.28	0.33	0.41	0.45	0.48	0.51	0.50	0.56	0.59	0.46
REG	3.68	3.13	5.22	6.52	5.91	6.20	5.00	7.19	6.12	5.44
**Av. RMSE**										
RTK	0.28	0.34	0.38	0.43	0.49	0.52	0.55	0.57	0.69	0.47
CLAS	0.27	0.34	0.40	0.47	0.51	0.51	0.56	0.62	0.65	0.48
REG	3.96	3.47	4.86	6.17	5.69	6.10	5.91	7.06	7.10	5.59

**Table 7:** Root Mean Squared Errors for yaw angles.

	Yaw (deg.)									
**Angle (deg)**	**5**	**10**	**15**	**20**	**25**	**30**	**35**	**40**	**45**	**Av. RMSE**
**Constant**										
RTK	0.41	0.53	0.69	0.82	0.94	1.02	1.15	1.05	1.26	0.87
CLAS	0.36	0.50	0.61	0.73	0.86	1.00	1.18	1.20	1.15	0.84
REG	5.10	6.15	8.00	9.60	9.32	11.00	15.80	18.60	14.90	10.94
**Sin**										
RTK	0.31	0.47	0.61	0.70	0.85	0.94	0.98	1.14	1.10	0.79
CLAS	0.34	0.49	0.59	0.77	0.88	0.97	1.01	1.07	1.09	0.80
REG	3.89	5.02	8.35	7.12	8.60	9.96	13.90	15.80	13.40	9.56
**Av. RMSE**										
RTK	0.36	0.50	0.65	0.76	0.90	0.98	1.07	1.10	1.18	0.83
CLAS	0.35	0.50	0.60	0.75	0.87	0.99	1.10	1.14	1.12	0.82
REG	4.50	5.59	8.18	8.36	8.96	10.48	14.85	17.20	14.15	10.25

## Data Availability

The raw data supporting the conclusions of this article will be made available by the authors on request.
